# InterPepRank: Assessment of Docked Peptide Conformations by a Deep Graph Network

**DOI:** 10.3389/fbinf.2021.763102

**Published:** 2021-10-25

**Authors:** Isak Johansson-Åkhe, Claudio Mirabello, Björn Wallner

**Affiliations:** Division of Bioinformatics, Department of Physics, Chemistry and Biology, Linköping University, Linköping, Sweden

**Keywords:** protein-protein interaction, machine learning, protein-peptide interaction, graph neural net, quality assesment

## Abstract

Peptide-protein interactions between a smaller or disordered peptide stretch and a folded receptor make up a large part of all protein-protein interactions. A common approach for modeling such interactions is to exhaustively sample the conformational space by fast-Fourier-transform docking, and then refine a top percentage of decoys. Commonly, methods capable of ranking the decoys for selection fast enough for larger scale studies rely on first-principle energy terms such as electrostatics, Van der Waals forces, or on pre-calculated statistical potentials. We present InterPepRank for peptide-protein complex scoring and ranking. InterPepRank is a machine learning-based method which encodes the structure of the complex as a graph; with physical pairwise interactions as edges and evolutionary and sequence features as nodes. The graph network is trained to predict the LRMSD of decoys by using edge-conditioned graph convolutions on a large set of peptide-protein complex decoys. InterPepRank is tested on a massive independent test set with no targets sharing CATH annotation nor 30% sequence identity with any target in training or validation data. On this set, InterPepRank has a median AUC of 0.86 for finding coarse peptide-protein complexes with LRMSD < 4Å. This is an improvement compared to other state-of-the-art ranking methods that have a median AUC between 0.65 and 0.79. When included as a selection-method for selecting decoys for refinement in a previously established peptide docking pipeline, InterPepRank improves the number of medium and high quality models produced by 80% and 40%, respectively. The InterPepRank program as well as all scripts for reproducing and retraining it are available from: *http://wallnerlab.org/InterPepRank* .

## 1 Introduction

Interactions between a short stretch of amino acid residues and a larger protein receptor, referred to as peptide-protein interactions, make up approximately 15–40% of all inter-protein interactions (Petsalaki and Russell, 2008), and are involved in regulating vital biological processes (Midic et al., 2009; Tu et al., 2015). These short peptides have a high degree of conformational freedom and can be part of larger disordered regions (Neduva, Victor et al., 2005; Petsalaki and Russell, 2008), making them difficult to study experimentally. However, knowledge of structural details such as interactions is crucial to understanding the molecular mechanisms underlying the interactions and to guide further experiments. Because of the inherent flexibility of the peptide fragments, computational prediction of the structural details of peptide-protein interaction complexes is challenging.

Several methods for predicting the structure of peptide-protein complexes exist, such as pepATTRACT (Schindler et al., 2015), CABSDOCK (Kurcinski et al., 2015), HPEPDOCK (Zhou et al., 2018), and PIPER-FlexPepDock (Alam et al., 2017). Template-based methods utilizing similarity to previously experimentally determined complexes, such as SPOT-Peptide (Litfin et al., 2019), GalaxyPepDock (Lee et al., 2015), and InterPep2 (Johansson-Åkhe et al., 2020a), have consistently shown high performance in previous benchmarks but are limited by available templates.

However, since the peptide ligand is a smaller molecule, it is possible to exhaustively sample the binding space by Fast-Fourier Transform docking (FFT docking). Classically, this requires a close to correct rigid receptor and ligand, but a set of poses derived from protein fragments with a sequence similar to the peptide can consistently produce conformations near the native bound conformation (Alam et al., 2017; Zhou et al., 2018). While the strength of FFT docking is that it allows for an exhaustive search of the docking space, the problem, as we will show, is that the energy function is a limited approximation of the binding affinity, and thus even though the method samples many near-native decoys it often fails to separate them from poor decoys. Additionally, rigid-body docking needs to be followed by much more computationally expensive refinement to reliably reproduce native contacts and binding modes.

A workflow previously shown to be successful is to rescore and refine promising decoys using a more advanced energy function, such as the Rosetta energy function (Raveh et al., 2010). Indeed, previous works have shown great success in the peptide-protein docking area by combining FFT-based docking with Rosetta refinement (Alam et al., 2017). However, because of the computational cost in running refinement, only a comparatively small subset of the FFT-generated decoys can be used. The selection of this subset is based on the energy function of the FFT method, thus several poor decoys will still go through refinement, even if they are unsalvageable. An improvement to this approach would be to run all decoys through a fast and accurate re-scoring algorithm to select decoys for refinement, rather than relying on energy functions constrained to FFT compatibility.

Many methods have been developed for the re-scoring of protein-protein complexes. For example: PyDock evaluates decoys based on pairwise electrostatic potentials and desolvation energy (Cheng et al., 2007; Pallara et al., 2017). Zrank and Zrank2 utilize van der Waals in excess of electrostatics and desolvation (Pierce and Weng, 2007, Pierce and Weng, 2008). DFIRE uses knowledge-based distance-dependent potentials with an ideal gas reference state (Zhou and Zhou, 2002). OPUS-PSP uses orientation-dependent packing and knowledge-based repulsive energy (Lu et al., 2008). SIPPER uses statistical residue-pair potentials derived from a curated interaction-set (Pons et al., 2011). Still, re-scoring methods are most often designed primarily for monomeric model quality assessment or protein-protein docking, and the methods with published use-cases on peptide-protein complexes to the best of our knowledge still rely on either physics-based, knowledge-based, or empirically tested energy function that do not take evolutionary information of the target into account. More advanced methods using machine learning, like ProQDock (Basu and Wallner, 2016b), are characterized by high computational costs that make them unfeasible for application to a large set of decoys, and they are better suited for evaluating a small set of refined models.

Within structural bioinformatics, Graph Convolutional Networks (GCNs) have seen increased use recently through applications such as PipGCN (Fout et al., 2017), which uses a pairwise classification architecture and evolutional features to predict protein-protein binding sites. Gligorijevic et al., 2019 utilized pre-trained LSTM sequence feature extraction to a graph network to classify protein function. EGCN uses edge-based graph convolutions to score protein-protein complexes with the use of simple features such as side-chain charge and hydrophobicity (Cao and Shen, 2019). GCNN encodes spatial information into the residue-nodes to classify proteins (Zamora-Resendiz and Crivelli, 2019). A Graph Convolutional Network avoids the spatial limitations of a classic convolutional network and allows for the direct definition of spatial relationships on a case-by-case basis. It passes information along pre-defined edges between nodes rather than by proximity in the input-matrix, while still allowing for complex information, such as evolutionary information, to be encoded in the nodes.

In this work, we present InterPepRank, a fast, novel, re-scoring algorithm utilizing GCNs to represent peptide-protein complex decoys with the added context of evolutionary information. InterPepRank is capable of quickly sifting through and ranking the complete space of conformations generated by FFT methods and improving the selection of decoys for subsequent refinement, thus improving the performance of ab-initio docking pipelines.

## 2 Materials and Methods

### 2.1 Metrics

Several different metrics are used to evaluate the performance of InterPepRank and other methods benchmarked.

#### 2.1.1 Metrics for Rigid-Body-Docked Decoys - Correct Decoys

In the perspective of ranking rigid-body-docked decoys, a decoy is defined as correct if it has the peptide positioned within 4.0Å ligand root-mean square deviation (LRMSD) of the native conformation (the experimentally determined structure of the complex), corresponding to DockQ scores of at least 0.28 (Basu and Wallner, 2016a). This limit was selected both as it is within the reported limit of when the Rosetta FlexPepDock refinement protocol can reliably refine decoys to sub-ångström precision (Raveh et al., 2010), and since it is below the CAPRI limit for an acceptable prediction of a docked peptide-protein complex (Lensink et al., 2017).

#### 2.1.2 Metrics for Refined Models - Acceptable, Medium, High

The CAPRI standard for classifying the model quality of peptide-protein complexes is used when assessing refined models, see Table 1. A model which is at least high quality will also be at least medium and acceptable quality, and a medium quality model will also be at least acceptable quality.

**TABLE 1:** CAPRI criteria for peptide-protein docked model quality (Lensink et al., 2017) and the equivalent values in terms of DockQ score (Basu and Wallner, 2016a). iRMSD is the root mean square deviation of residues at the native interface; fnat is the fraction native residue-residue contacts recalled.

*Model Quality*	LRMSD	iRMSD	Fnat	DockQ
Acceptable	< 5.0 Å	< 2.0 Å	> 0.2	> 0.23
Medium	< 2.0 Å	< 1.0 Å	> 0.5	> 0.49
High	< 1.0 Å	< 0.5 Å	> 0.8	> 0.80

#### 2.1.3 ROC

A receiving operand characteristic-curve (ROC curve) measures how two metrics (here the False Positive Rate (FPR) and the True Positive Rate (TPR)) change in relation to each other as the threshold for scoring is varied. The False Positive Rate is defined as: *FPR* = *FP*/*N*, where *FP* is the number of decoys incorrectly identified as correct and *N* is the total number of incorrect decoys in the set. The True Positive Rate is defined as: *TPR* = *TP*/*P*, where *TP* is the number of correctly identified correct decoys and *P* is the total number of correct decoys in the set.

ROC curves are especially suited to comparing sets of varying difficulty to each other, since the curves are unaffected by class imbalance. In this case, this allows us to compare method performance on decoy sets from peptide-protein pairs with varying ratios of correct-to-incorrect decoys in the same test. However, as argued by Saito and Rehmsmeier (2015), ROC curves fail to accurately depict the absolute performance of predictors, which is better described by precision-recall curves. As such, for every ROC curve a corresponding precision-recall curve can be found in the Supplementary Information.

### 2.2 IPR0220 Dataset

A set of 6,857 interacting peptide-protein pairs taken from the Protein Data Bank (PDB) (Berman et al., 2000) at October 15, 2018. A peptide-protein interaction is defined as a peptide of 25 or fewer residues sharing a contact surface of at least 400Å^2^ with a receptor of 50 residues or more. The interface size requirement was included to ensure the set contained only complexes with direct interaction between the peptide and receptor. In accordance with previous analysis in Johansson-Åkhe et al. (2018), this interface size represents roughly five residues in direct interaction. The set was then redundancy-reduced at 30% sequence identity down to 687 representative target pairs for testing and validation, with the other 6,170 targets being reserved for training set augmentation.

#### 2.2.1 Decoy Set Generation

For each of the initial 6,857 target pairs (containing redundancy), possible docking poses were generated in the following way: 50 different peptide conformations were generated using the Rosetta fragment-picker (Gront et al., 2011) using local sequence similarity of the peptide. Each peptide conformation was exhaustively docked on the surface of its receptor by PIPER (Kozakov et al., 2006) using FFT docking. 70,000 decoys were generated for each conformation for the 687 representative pairs to be used as testing and validation data, and 4,375 for each conformation for the 6,857 pairs used in training. In total, almost 2.5 billion (687 × 50 × 70,000) decoys were generated for testing. The full test data set is available online (Johansson-Åkhe et al., 2020b).

#### 2.2.2 Splitting of Representative Pairs Into Partial Sets

The set of 687 target pairs was split into 14 partial sets of approximately 50 target pairs each, with the requirement that no two receptors in the same partial set could be related. Here we define two receptors as “related” if they share the same CATH superfamily (Dawson et al., 2017) or if they secure a TM score >0.4 (Zhang and Skolnick, 2005), the latter applied to the cases where at least one of the two receptors lack CATH annotation. This cutoff was chosen as it is a previously established limit of correct alignments between homologous proteins (Zhang and Skolnick, 2004).

One of the 14 partial sets was randomly selected and set aside as the validation set. The network parameters and architecture were optimized on the validation set once, while early stopping was performed separately with the help of the validation set for each training set. No parameters or features were ever optimized with regard to performance on any of the other partial sets. To enable rapid method development, the number of decoys per target in the validation set was limited to a random subset of 2,500 decoys.

#### 2.2.3 Training and Test Sets

After reserving one of the 14 partial sets for validation purposes, we performed cross-fold testing of the remaining 13 partial sets. This means that each of these 13 sets was used as test set once. For each test set we then had an initial training set that was made of the other 12 partial sets (not including the validation set). Then, in order to maximize the amount of training data, we augmented the training set by adding from the original (non-redundancy-reduced) dataset of 6,857 only those targets that were not ‘related’ (as defined above) to, or shared more than 30% sequence identity with, any target in the set currently used as the test set or the validation set. Since the number of incorrect decoys generally outweighed the number of correct decoys, we further filtered out decoys to make sure that each target pair contributed only as many incorrect decoys as correct decoys. Additionally, to avoid large CATH superfamilies contributing disproportionate amounts of decoys, which would bias a training set, the number of decoys for each CATH superfamily was limited by the median number of decoys contributed across all CATH superfamilies. As a result of this selection, each peptide-protein pair ended up contributing on average 426 decoys divided equally between correct and incorrect decoys. In all, each of the training sets consisted of on average 1,048,386 decoys. Once more, although a new training set was generated for each test set, the neural network parameters and hyperparameters were never optimized with regard to performance on any test set, but always and only with regard to performance on the validation set. Supplementary Figure S1 provides a graphical explanation of the test, validation, and training set construction.

#### 2.2.4 Expanded Analysis Set

One of the test sets was randomly selected as an Expanded Analysis set for running additional analysis and tests. In particular it was used to run computationally intensive refinement methods to benchmark the final impact of re-scoring on full docking protocols, and comparisons to relatively slow re-scoring methods such as pyDock3.

#### 2.2.5 APO/HOLO Set

In most real-world docking scenarios, the experimental structure of the bound receptor is unknown; often the receptor in question has not been structurally determined with a binding partner at the same interface before, in which case, the unbound (APO) form of the receptor without the peptide ligand, or even a predicted structural model of the receptor, has to be used. Unfortunately, compared to the number of bound complexes with known structure, the number of proteins with experimentally determined structures both with and without a peptide ligand bound is very low, resulting in few unique examples for testing and training. Thus, many methods, among them InterPepRank, are trained starting from bound (HOLO) structure of the receptors, sometimes limiting their performance in real-world cases if they failed to generalize. A conscious design choice was made with InterPepRank to counteract this potential failure by not including any high-resolution structural features, such as side-chain orientation. Similarly, the low resolution of the features should eliminate the need to explicitly model or add solvent or hydrogen atoms, which are not modeled by FFT docking methods such as PIPER or ZDOCK.

To test the success of this design choice and the generalizability of InterPepRank to real-world cases, an APO/HOLO set of docked decoys was constructed from a list of annotated protein-peptide-binding complexes for which both the APO and HOLO structures of the receptor are known, generously provided to us by the AutoPeptiDB (London et al., 2010) developers. The set was redundancy-reduced on CATH superfamily annotation, yielding 56 peptide-protein complexes, and decoys were generated for each HOLO and APO complex in the same way as for all cases in the test sets. The data is published here (Johansson-Åkhe et al., 2020b).

### 2.3 Representation and Architecture

The scoring of a decoy with InterPepRank is performed by a multi-layer neural network using graph convolution layers as a main component.

#### 2.3.1 Decoy Representation

The graph network utilizes edge-conditioned graph convolutions, where the input is a set of nodes, each described by a set of input features, as well as weighted undirected edges between nodes. In this case, nodes represent individual residues of a decoy and edges denote different types of interactions between residue pairs, the types are specified under Edge Features below.

#### 2.3.2 Node Features

Here, the node features used were amino acid code (one-hot encoded, 21 values to account for unknown residues, meaning a binary/bit vector of length 21 where in this case any single value being assigned as 1 represents a specific amino acid at that position), a Position-Specific Scoring Matrix (21 values including gaps, Eq. 1), Self-entropy (21 values, including gaps, Eq. 2), and one variable denoting if the residue belongs to the peptide or receptor (1 value), for a total of 64 features. Multiple sequence alignments for calculation of PSSM and self-entropy were acquired by running two iterations HHblits (Remmert et al., 2012) 2.0.15 against uniclust30_2016_03, with a maximum pairwise sequence identity of 90% and an E-value inclusion threshold of 0.001. Peptide fragments are generally too small to produce a meaningful multiple sequence alignment, and since we cannot assume that all peptides are fragments of larger protein sequences, the PSSM and Self-entropy features are set to 0 for the peptide residues. If these features could be included, we would expect a rise in performance assuming the network could be re-trained with these features for most complexes in the training sets.

$$PSSM_{i}=-\log\frac{p_{i}}{p_{bi}}$$ (1)

$$S_{i}=-p_{i}\log\frac{p_{i}}{p_{bi}}$$ (2)

where *p*_*i*_ is the frequency of the amino acid on position *i* recurring at that position in the multiple sequence alignment and *p*_*bi*_ is the background probability of that amino acid.

#### 2.3.3 Edge Features

Four types of one-hot encoded edges were used: self-edges to allow the passing on of information to the same residue in consecutive layers, sequence edges denoting the existence of a peptide bond between two residues, proximity edges between each pair of residues with any heavy atoms within 4.5Å, and lastly an edge feature to summarize the identity of an edge is set to one where any other edge feature is true. The identity edge speeds up calculations through filtering where convolutions need to be performed. For each decoy, up to 100 residues or nodes were used, consisting of the peptide and the residues of the receptor closest to the peptide, i.e., if the peptide were 25 residues long and the receptor 170 residues, the 100 nodes would include the 25 peptide residues and the 75 residues from the receptor closest to the peptide. Complexes with fewer than 100 residues are zero-padded. Since only 100 nodes maximum are considered, it is possible that the whole interface is not captured by the representation in the case of a large buried peptide. However, an analysis on the validation data revealed that the full interface of every decoy could be captured by the 100 node limited representation.

#### 2.3.4 Target Function

To facilitate training, the raw LRMSD values were normalized to the [0,1] range using the same normalization scheme as in Levitt and Gerstein (1998):

$$LRMSD_{\mathrm{norm}}=\frac{1}{1+{\left(\frac{LRMSD}{4.0}\right)}^{2}}$$ (3)

Since networks are often observed to learn quicker from classification tasks, the problem was formulated as a classification problem by predicting to which bin of *LRMSD*_norm_ a decoy belonged to. Different networks were trained to classify between two to four classes evenly spread in the [0,1] range. Class probabilities were then used to calculate a final predicted score, *S*, using a sum of the *LRMSD*_norm_ bins weighted by the predicted probabilities:

$$S=\underset{i}{\sum }x_{i}P\left(x_{i}\right)$$ (4)

where *i* is the number of bins, *x*_*i*_ the center of bin *i* and *P*(*x*_*i*_) the predicted probability for bin *x*_*i*_.

#### 2.3.5 Network Architecture

**FIGURE 1:**
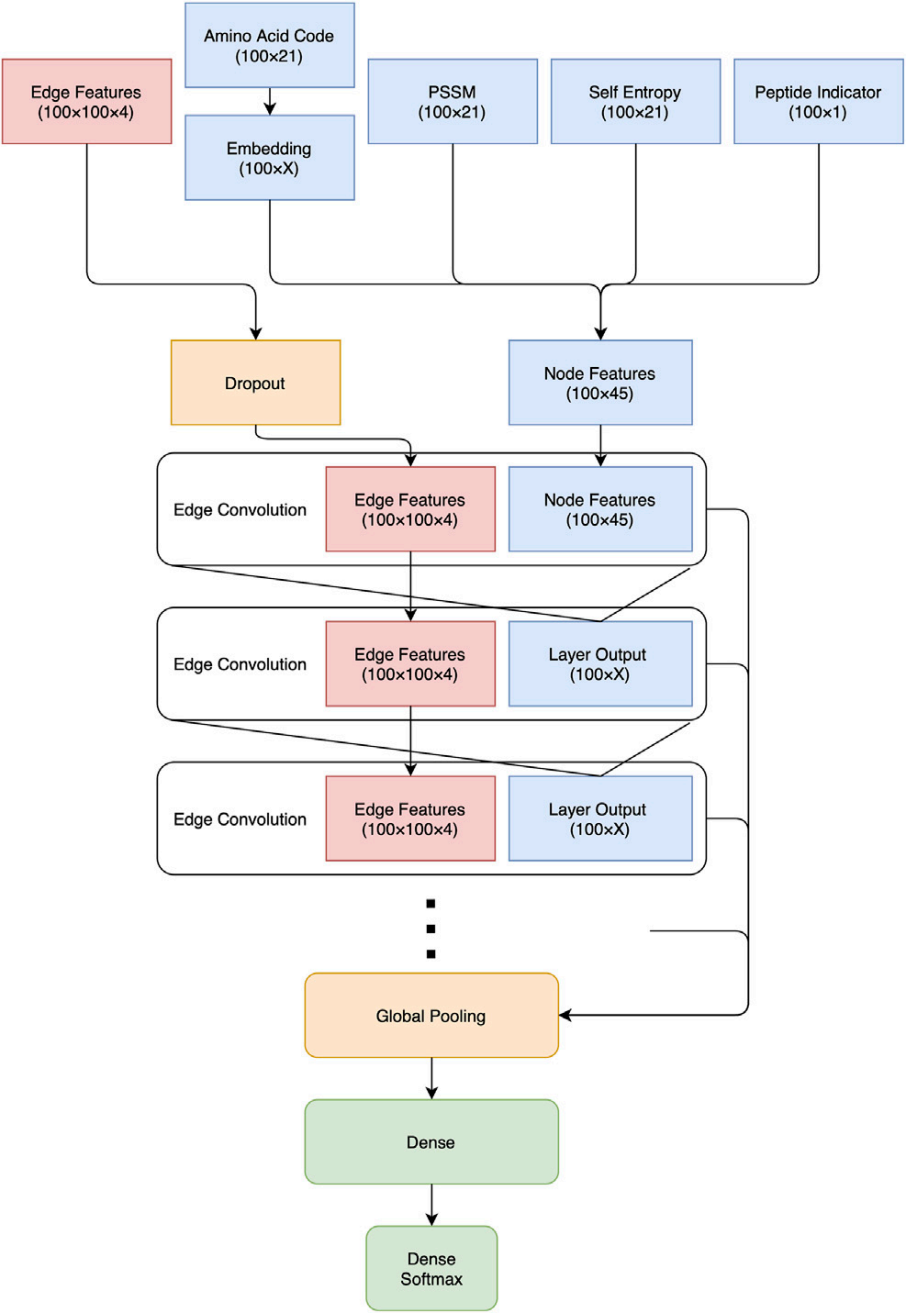
The basic architecture of the InterPepRank networks. Note that the output from the Edge Convolution and Embedding layers have been denoted as X, since different values were sampled. Throughout the net, the ReLU activation function is applied after each convolution.

The network was implemented as a feed-forward graph convolutional network, see Figure 1. Before any convolutions were applied, the node feature amino acid code (one-hot encoded 21 value feature) was passed through an Embedding Layer to reduce dimensionality and thus also the number of weights, limiting overfitting. Embedding layers are small network architectures for mapping discrete labels onto continuous space, and are frequently used in areas such as language processing (Mikolov et al., 2013), and have previously been successfully used to describe amino acids (Mirabello and Wallner, 2019).

Next, the node features were passed through a varied number of Edge Convolution Layers (Simonovsky and Komodakis, 2017), with ReLU activation between each layer, taking the output of the previous layer as node features for the next, while keeping the same edge features throughout. Edge Convolution Layers learn filters as a function of both node and edge features, and apply these filters along the edges of the graph.

The output from each convolution layer was concatenated together before global pooling of all node features, followed by two dense layers before prediction. To improve the robustness of the network to noisy or incomplete data and help it generalize, a dropout ranging between 0.1 and 0.25 was applied to the edge features, meaning 10–25% of all edges were set to 0 for each decoy. Different sizes of the Edge Convolution and Dense outputs were explored, as well as different methods for pooling, see below.

The network was implemented using Spektral and Keras (Chollet et al., 2015) with the layers proposed by Simonovsky and Komodakis (2017), and trained with the TensorFlow backend (Abadi et al., 2015).

#### 2.3.6 Parameter Optimization and Model Selection

Parameters and hyperparameters of the networks were optimized to maximize performance on the validation set. Additionally, to increase the predictive power of InterPepRank with little impact on runtime, some of the best-performing trained networks were ensembled by averaging their outputs.

Although different learning rates and optimizers were explored on the validation set, the final networks were trained with an adaptive moment estimation (ADAM) optimizer with a learning rate of 0.001 for a maximum of 1,000 epochs. The weights from the epoch with the best combination of Spearman rank-order correlation, loss, and precision/recall on the validation set were selected as final weights.

## 3 Results and Discussion

In this work we have developed InterPepRank, a machine learning-based method which encodes the structure of a peptide-protein complex as a graph; with physical pairwise interactions as edges and residue information including evolutionary features such as PSSM and sequence conservation as nodes. The graph representation is trained to predict the LRMSD of decoys on a large set of peptide-protein complexes. Different network architectures were tried and the nine best (0–8) predictors at validation time are shown in Supplementary Figure S2, see Supplementary Information for details of specific network architectures. To maximize performance, a subset of these were averaged in an ensemble predictor, and the best ensemble used all networks except numbers 5 and 6.

### 3.1 Ablation Study

**FIGURE 2:**
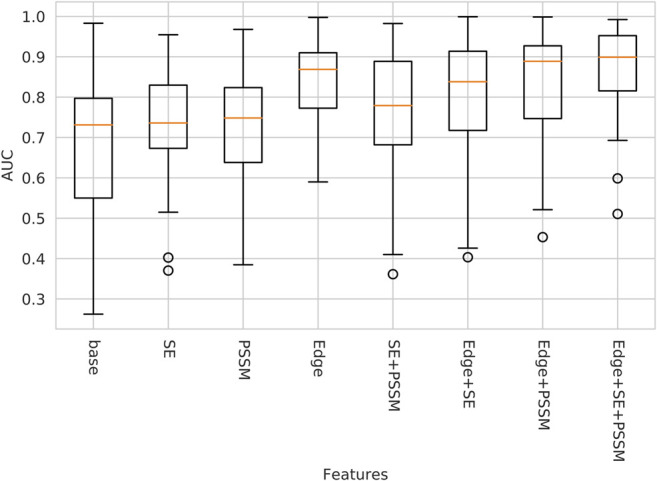
AUC on validation data using different subsets of all features. Edge denotes the edge identities, and the lack of the Edge features means that only proximity is considered. PSSM denotes the position specific scoring matrix. SE denotes self-entropy.

An ablation study was performed on the validation data to analyze which features contributed most to performance. Starting from a *base*, consisting of only the amino acid code and peptide indicator as node features and the proximity edge as the only edge feature, more information was successively added in a step-wise fashion. The performance was measured by the ROC AUC, see Figure 2. The additional features of *Edge* (consisting of differentiating between covalent edges, self-edges, and simple proximity edge), Self-entropy (*SE*), and *PSSM* where included individually and in combinations. The single most powerful feature was *Edge*, while the inclusion of Self-entropy and PSSM showed minor increases in performance. This is seemingly contrary to our initial hypothesis that the greatest performance increase would stem from the inclusion of evolutionary information. However, with a varied enough training set such information can be inferred directly from the sequence, which is included in the *base* network. Methods to infer features from sequence have previously been used to avoid generating multiple sequence alignments in for example Hurtado et al., 2018.

Interestingly, in the case of *Edge + SE* the inclusion of Self-entropy seems to have had a negative impact on the performance, and adding only Self-entropy to the base network seems to have had little to no effect on the median AUC. However, this trend is not observed if *PSSM* is also used, in which case performance is always improved, indicating that knowledge of interface conservation alone is not sufficient but needs the evolutionary context of the *PSSM*.

For a further analysis of the differences in the performance of the different network architectures see Supplementary Information.

### 3.2 Comparison to Established Methods

To put the performance of InterPepRank into perspective, we compared its performance to several established state-of-the-art scoring methods: PIPER (Kozakov et al., 2006), pyDock3 (Cheng et al., 2007), DFIRE (Zhou and Zhou, 2002), and Zrank (Pierce and Weng, 2007), all tested on the same test sets as InterPepRank. PIPER was run with the same rotation and energy matrices as when part of the peptide-protein docking protocol PIPER-FlexPepDock (Alam et al., 2017). Both pyDock3 and Zrank were created for the scoring of protein-protein complexes, and DFIRE was originally devised as a monomer model quality assessment and stability predictor; however, pyDock3 was recently shown to efficiently identify near-native peptide decoys in the sixth CAPRI edition (Pallara et al., 2017), Zrank is included as one of the currently leading fast available protein-protein scoring functions according to recent benchmarks (Moal et al., 2013; Yan and Huang, 2019), and DFIRE has been shown to accurately predict peptide-protein binding affinity as part of the SPOT-Peptide protocol (Litfin et al., 2019). Despite being developed primarily for other purposes, PIPER, DFIRE, and pyDock3 have all been utilized by their developers for peptide-protein complex evaluation without any modifications to the methods. It should be noted that PIPER was used to generate the decoys of this study.

As discussed previously, although the aforementioned methods have been shown to work well with peptide-protein complexes, there is a lack of ready-to-use scoring functions developed specifically for peptide-protein complexes. Technically, the Rosetta FlexPepDock protocol (Raveh et al., 2010) can be run in a mode which only applies its scoring function without changing the structure. However, Rosetta utilizes a fine-grained scoring algorithm, and the structures need to be minimized using the Rosetta relax protocol to be scored properly.

**FIGURE 3:**
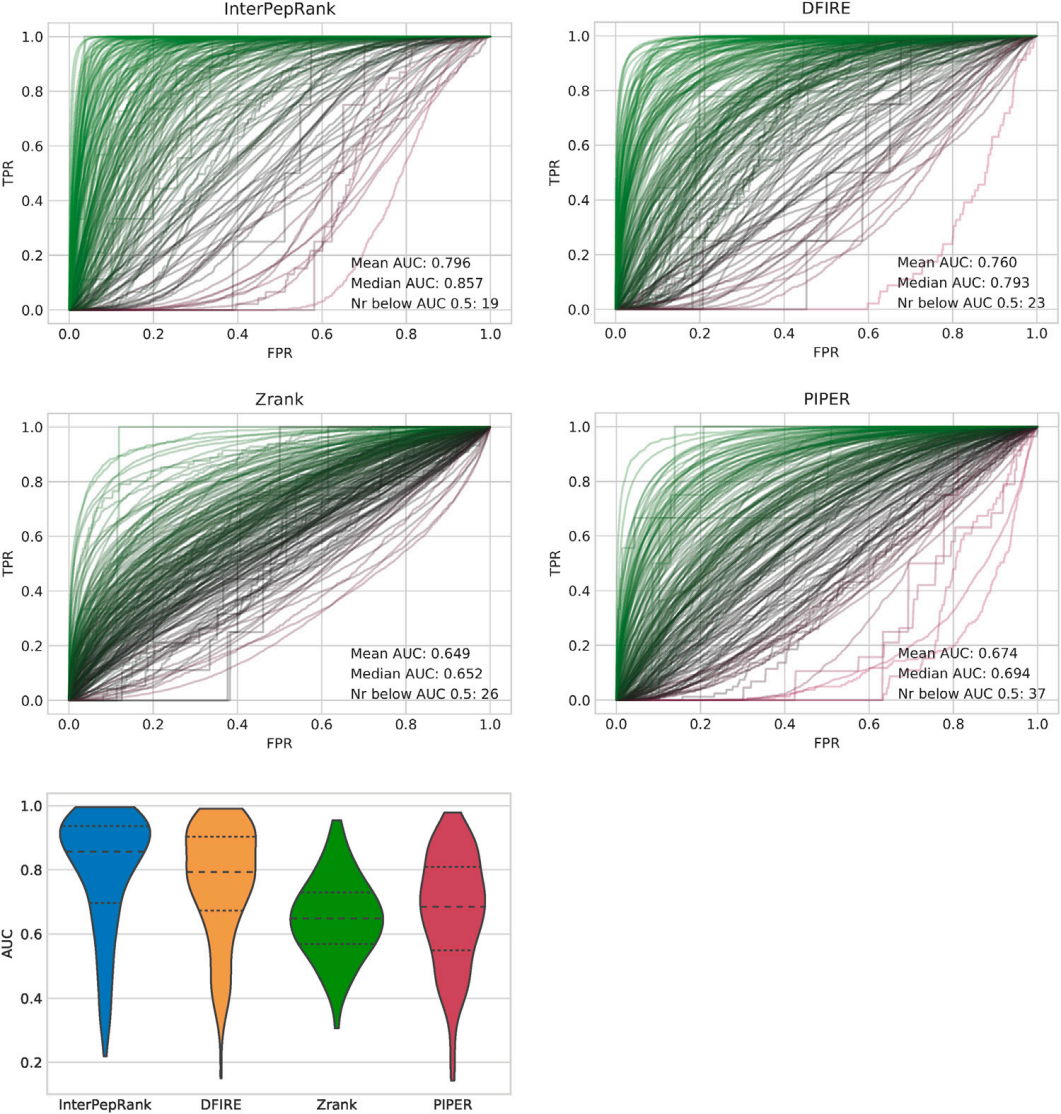
ROC-curves for the different methods, each target is represented by one curve, and a violin-plot over the distributions of AUCs. The area under the curve (AUC) displayed in the graphs is the average and median over all targets. Note that while the standard deviation of AUC is higher for InterPepRank, it still only has 19 targets with an AUC below 0.5, compared to 35, 37, and 26 targets for DFIRE, PIPER, and Zrank, respectively.

**FIGURE 4:**
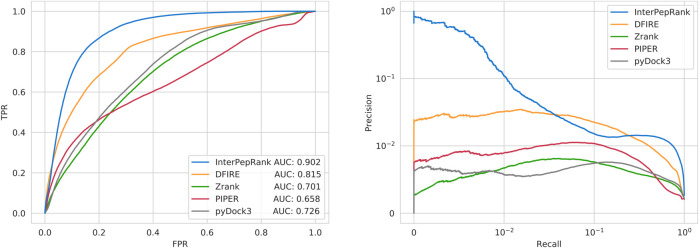
ROC-curves for using the method scores to separate low-LRMSD decoys from other decoys **(left)**, and Precision-Recall curves for the same thing **(right).** Note that the scale of the right curve is logarithmic, which is necessary because of the large class-imbalance of the problem. Zrank, DFIRE, pyDock3, and PIPER are normalized by the total length of receptor and peptide. Analysis run only on the decoys from the Expanded Analysis set.

The primary objective of this study was to develop a method for selecting decoys for further refinement, which means the metric of interest is the ability to rank decoys for individual targets. This was measured using the AUC from ROC curves for individual targets, see Figure 3. InterPepRank has a higher average AUC compared to all other tested methods and the distribution of AUC values is in general shifted toward higher values. The median AUC is 0.86 for InterPepRank compared to 0.79, 0.65, and 0.69 for DFIRE, Zrank, and PIPER, respectively, similar to the overall AUC for (Figure 4). The same trend holds true against pyDock3 and Rosetta FlexPepDock scoring as well on the Expanded Analysis set, see Supplementary Figure S5. Note, however, that there are some targets for which InterPepRank has a particularly low ROC AUC (lower than 0.3), something which for instance Zrank never has. This means that even though InterPepRank achieves higher performance on average, there are some targets for which it fails to generalize completely. Since Zrank appears more stable, and is particularly fast to run, perhaps a meta-predictor utilizing both scoring methods can be developed to account for this shortcoming in the future.

Another metric of interest is the ability to rank decoys even between targets, i.e., if the methods are capable of absolute decoy ranking. This was tested on the Expanded Analysis set using overall ROC and precision-recall curves, see Figure 4. Different ways to normalize for complex size for DFIRE, Zrank, pyDock3, and PIPER were tried and the method resulting in the best performance was selected. Overall, InterPepRank performed better than DFIRE, Zrank, pyDock3, and PIPER at assessing the absolute quality of individual decoys in comparison to both other decoys from the same target and between targets. The AUC for InterPepRank is 0.90 compared to 0.82, 0.73, and 0.70 for DFIRE, pyDock3, and Zrank, respectively. PIPER is slightly worse with AUC 0.66. In fact, it is only InterPepRank that is able to achieve a useful precision (>0.1) for its highest ranking prediction. This might be expected since the other scoring methods assess the whole complex and not only the peptide interaction. The overall AUC rankings in Figure 4 mirror the median individual AUCs in Figure 3. This indicates that the methods tested should be able to pick up refineable decoys from a pool of different receptors and ligands at roughly equal efficiency as they rank decoys internally, implying the results from the tests focusing on internal ranking could be generalized to an overall selection case, and InterPepRank can be used for cross-target comparable scoring.

#### 3.2.1 Runtime Analysis

Although slower than Zrank and DFIRE, InterPepRank still operates well within the realm of possibility for large-scale studies, see Table 2. It should be noted that most of the time (>95%) of InterPepRank is spent preparing the graph-input. This is currently implemented in a Python script but could be optimized as compiled code.

**TABLE 2:** Average runtime for 70,000 decoys over all targets, as measured in minutes. All calculations were performed on a single CPU core of Intel Xeon Gold 6130 running on CentOS 7, and when applicable an Nvidia GeForceRTX 2080 Ti graphics card was available. If InterPepRank is run completely on CPU, add approximately 30 min to the mean runtime. The average receptor size of the set is 156 residues.

Method	Mean runtime	60 res. Receptor	472 res. Receptor
InterPepRank	100.6	57.2	249.2
Zrank	9.2	2.9	24.7
pyDock3	664.4	293.1	1,372.6
DFIRE	7.6	4.0	35.5
Rosetta FPD scoring	590.0	256.6	1,624.5

### 3.3 Disordered Peptides in the Dataset

To analyze whether InterPepRank shows any bias toward peptides which are disordered when unbound, all peptide fragments in the extended analysis dataset were investigated with the DISOPRED program (Ward et al., 2004), which predicts disorder on a residue-by-residue basis. In total, 11.6% of all peptides in the test sets were predicted to be disordered when unbound (in this case, the peptide is considered disordered if at least 75% of its residues are predicted as disordered). The difference in InterPepRank AUC between ranking decoys where the peptides were predicted as disordered and those where less than 10% of the peptide was predicted as disordered cannot be said to be significant (*p*-value > 0.5, Supplementary Figure S4), meaning InterPepRank sees no increased difficulty in ranking peptides which are disordered when unbound. For all the other methods investigated in Figure 3 however, the difference in AUC is significant with *p*-values < 0.04, indicating such peptides pose a problem for these methods.

Additionally, the specific peptide residues predicted as most likely to undergo disorder-to-order transitioning upon binding as predicted by Proteus (Basu et al., 2017) were investigated in the top 100 decoys for each complex as ranked by the different methods. Among the top decoys of InterPepRank, there is a bias in favor of placing residues predicted to undergo disorder-to-order transitioning at the interface (R = 0.33, *p*-value = 0.006), which is not seen in the other methods investigated (R < 0.09, *p*-values > 0.336), except for PIPER, which also shows a slight preference for these residues, but with weaker correlation (R = 0.21, *p*-value = 0.085). The version of PIPER used here is specifically tuned for peptide-protein interactions by focusing on the core binding motif of peptide fragments (Alam et al., 2017). As such, it is unsurprising that it manages to identify regions responsible for binding in the longer peptides in this dataset with at least some correlation.

### 3.4 Proof of Concept: Improving the PIPER-FlexPepDock Pipeline

The purpose of InterPepRank is to provide an accurate re-scoring step for selecting which decoys from FFT-based rigid-body-docking are worth further refining to achieve sub-Ångström docked complexes. The PIPER-FlexPepDock pipeline (Alam et al., 2017) uses FFT-based PIPER to dock peptides of varying conformations onto a receptor surface, and the PIPER score to select 12,500 decoys for further refinement by the Rosetta FlexPepDock protocol, followed by clustering the top 1% of the refined decoys to make final docking predictions.

As a proof of concept, the full PIPER-FlexPepDock pipeline was run on the Expanded Analysis set both as is, and with InterPepRank, Zrank, DFIRE, or pyDock3 instead of PIPER for selecting decoys for refinement. The results were analyzed according to the CAPRI standard of evaluating the quality of peptide-protein complex models, see Table 3. It should be noted that many of the peptides in the Expanded Analysis set are longer than the longest peptides investigated in the original PIPER-FlexPepDock benchmark, explaining the overall decrease in performance as compared to that study. From Table 3 it can be seen that InterPepRank, DFIRE, and Zrank improve the performance of PIPER-FlexPepDock by selecting better decoys for refinement. While using DFIRE and Zrank also lead to a higher number of acceptable models, using InterPepRank leads to a higher yield of both medium and high quality models. This is in line with the ROC curves in Supplementary Figure S5 where InterPepRank has a higher average and median AUC, but Zrank has fewer decoys with poor ROC AUC below 0.5. It is also reflected in the overall distribution LRMSD of the decoys selected for refinement by InterPepRank and Zrank: while InterPepRank both in mean and median selects decoys with lower LRMSD, Zrank prefers a wider sampling of conformations resulting in a generally smaller number of low LRMSD decoys, but more targets with at least one low LRMSD decoy, see Supplementary Figure S6.

**TABLE 3:** For how many of the targets in the Expanded Analysis set the modified PIPER-FlexPepDock pipeline produced models of the different quality measures among the top 10 results. As Rosetta FlexPepDock is a Monte-Carlo based approach, the part of the pipeline utilizing the FlexPepDock protocol and onwards was run in triplicates and the results averaged. Note that Acceptable means any model of at least acceptable quality (same for Medium). Highest number per column marked in bold.

Re-scoring method	Acceptable	Medium	High
InterPepRank	15.33	**9.66**	**3.33**
DFIRE	16.33	7.0	1.33
Zrank	**18.0**	7.0	2.33
pyDock3	13.33	5.0	0.0
None (PIPER)	13.66	5.33	2.33

This test was conducted only on the Extended Analysis set, which is only one of the 13 dataset splits. As InterPepRank has fewer targets with AUC below 0.5 on the full test set, we hypothesize that in the general case using InterPepRank to select decoys for refinement would be superior also in generating acceptable models, as the larger full test set should be more representative of most real-world cases.

#### 3.4.1 Improving Runtime With Score Threshold

InterPepRank score is independent of complex size and composition, as it only depends on the absolute quality of each decoy, which can be seen in Figure 4. Because of this, it should be possible to introduce a score threshold, rather than simply always selecting the top 12,500 decoys for refinement, to avoid refining guaranteed bad decoys as predicted by the InterPepRank score.

Indeed, using an InterPepRank score cutoff of 0.47 to select decoys for refinement reduces the median number of decoys refined per target to 10,000 while maintaining performance. With this cutoff, at least one high or medium quality decoy will be produced for each target for which one was produced with the top 12,500 decoys, and 95% of all targets which had at least one acceptable decoy among the 12,500 will still have at least one among those scored over the threshold.

Considering the median runtime of FlexPepDock refinement being 1.55 min per decoy on the same systems used for the re-scoring method speed benchmark, this results in the usage of an InterPepRank threshold saving on average 3,875 min of runtime, making up for the extra time spent running InterPepRank.

### 3.5 Generalizability

The ultimate goal of any machine learning approach is to be able to generalize to new unseen examples and to avoid potential biases. By using a carefully designed testing strategy we have avoided any potential biases in the testing. However, two potential biases remain: we only used one docking method to generate all docking poses and the method is trained on bound docking cases.

#### 3.5.1 Docking Method Bias

The potential bias of InterPepRank toward decoys generated using PIPER, which was used to train InterPepRank, compared to decoys generated by ZDOCK (Pierce et al., 2014) and FMFT dock (Padhorny et al., 2016) was explored on the Expanded Analysis set.

Decoys were generated for the same receptor structures and peptide conformations as with PIPER for both ZDOCK and FMFT dock, using the default settings. Next, to ensure a fair comparison of the bias of InterPepRank toward decoys generated by different methods rather than comparing the actual performance of the docking methods, the quality of the decoys from the methods was balanced in such a way that for each target, each method contributed with an equal number of decoys for every 0.2Å LRMSD bin.

As can be seen in Supplementary Figure S7, there is a slight consistent difference in prediction error for the different methods. This bias is small (average error difference for decoys of similar LRMSD <0.01), consistently in the same direction for each method, and the predictive power of InterPepRank is not significantly different for any of the individual methods (*p*-values > 0.59). Because of this slight bias, mixing input structures to InterPepRank from different docking algorithms could hamper the predictive performance between decoys from different methods. However, as long as all input decoys are produced from the same algorithm, i.e., the pool of decoys being scored and ranked is not a mix of decoys created by different docking algorithms, the predictive power of InterPepRank should not differ significantly from the results reported in this paper as long as the decoys are sterically possible.

#### 3.5.2 APO/HOLO Docking

To test the generalizability to cases starting from an unbound receptor structure, InterPepRank was also run on the decoys generated from the APO/HOLO set. Care was taken to ensure that no decoy was scored by a network which had been trained on receptor structures sharing a CATH superfamily annotation with the receptor of the decoy.

**FIGURE 5:**
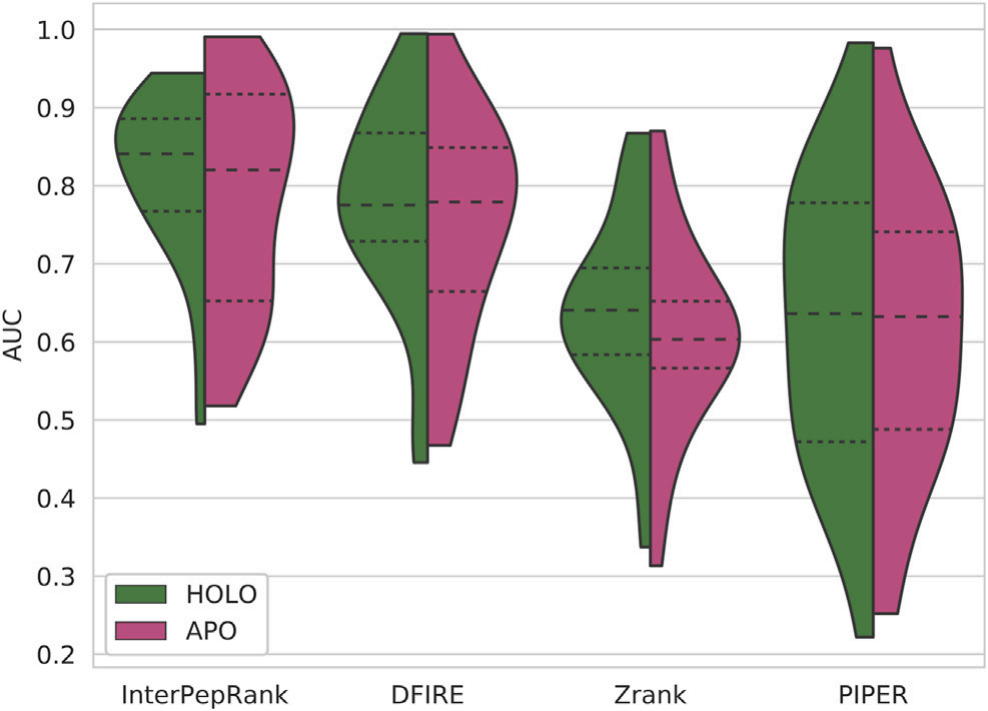
The ROC AUC distribution of the different scoring methods on the APO/HOLO Set. The HOLO halves of the violin-plots are the AUC distributions with the receptors in their bound states during scoring. The APO halves are the AUC distributions with the receptors in their experimentally determined unbound states.

The performance of the different methods tested on this set can be seen in Figure 5. All methods tested either suffered from a loss in median AUC or an increase in variance on AUC when tested on decoys generated with APO receptors as compared to the decoys generated with HOLO receptors of the same complexes. Additionally, even though InterPepRank only has access to low-resolution features, its performance still suffers from not having been trained on decoys generated with APO receptors. However, the performance of InterPepRank is still favorable compared to the other methods investigated, indicating that although the problem itself is harder, InterPepRank is generalizable enough compared to other methods that its usefulness for its intended purpose is not diminished.

### 3.6 Examples

As seen in previous works, machine learning methods for prediction of binding sites tend to predict protein-protein binding sites as potential peptide-protein binding sites, often resulting in difficulties in differentiating an interaction with the correct binding site from an interaction with another site, like one for protein-protein binding, or even crystal contacts (Johansson-Åkhe et al., 2018; Johansson-Åkhe et al., 2020a). Without the multi-layer inference of a trained machine learning model however, methods based purely on shape complementarity or electrostatics struggle to describe certain binding modes and can often be sensitive to small variations in decoy generation, such as how close together the receptor and peptide are placed (Basu and Wallner, 2016b; Mirabello and Wallner, 2017). Additionally, the non-machine learning based methods tested in this study perform significantly worse for ranking decoys where the receptor has false positive binding sites with high potential contact order (*p*-value < 0.02, Supplementary Figure S8), indicating a bias toward selecting decoys binding to *α*-helices or deep interfaces.

**FIGURE 6:**
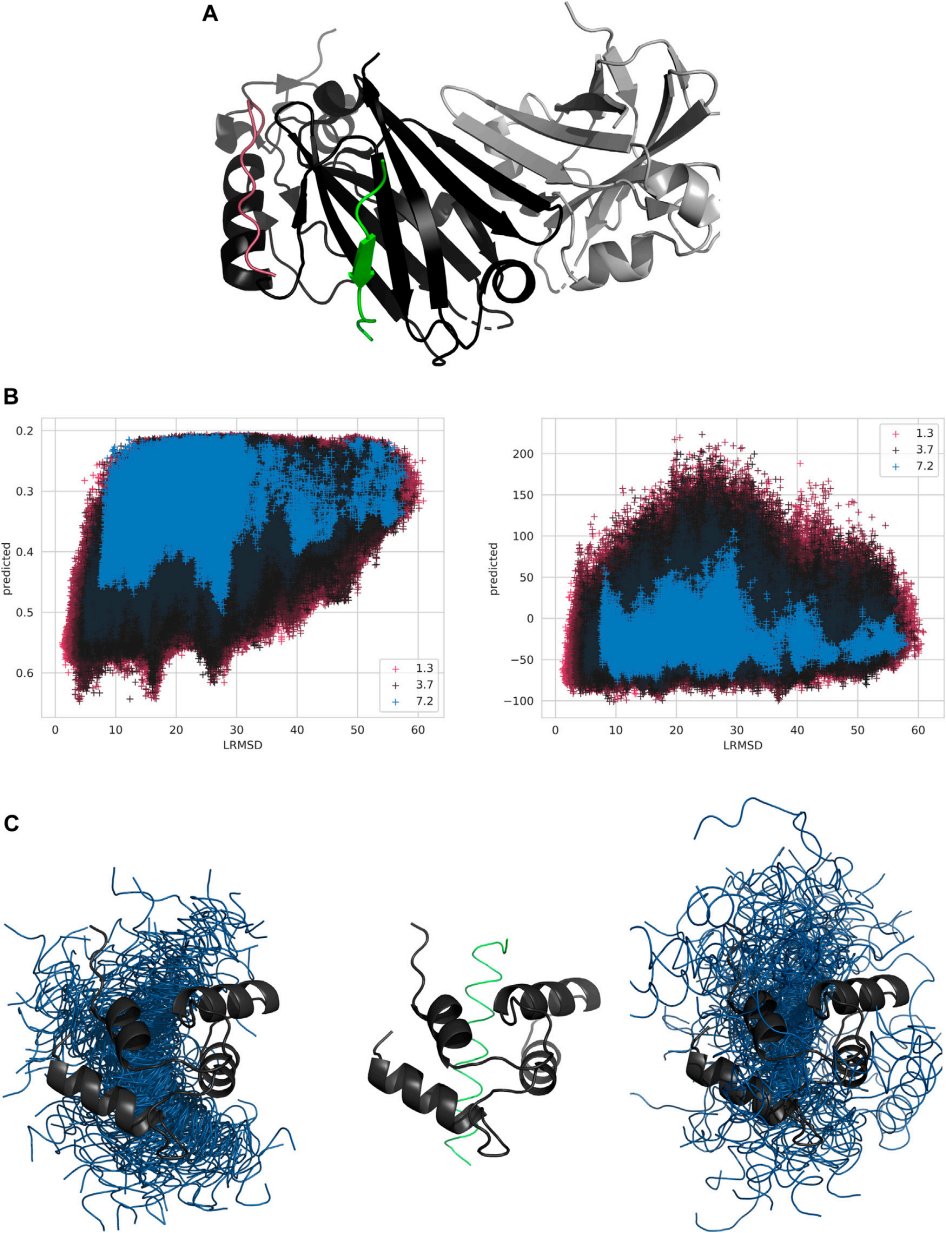
**(A)** Siah1 in complex with a synthetic peptide (PDB-ID 4i7b). Siah1 is shown in black, the native conformation of the peptide is shown in green, the other chain in the asymmetric unit is shown in gray, and an example peptide conformation maximizing interaction in the alternate groove is shown in pink. **(B)** Scatterplot of InterPepRank predicted score **(left)** and Zrank predicted score **(right)** versus LRMSD for all decoys of 4i7b chains A (receptor) and B (peptide). Each decoy is colored by the backbone RMSD of the peptide to its native conformation if superimposed. **(C)** C-terminal domain of Akazara scallop troponin C with fragment of troponin I (PDB-ID 3izt). Troponin C shown in black, native conformation of the peptide shown in green, and decoys generated by docking shown in blue. Left image shows the 100 top ranked decoys by InterPepRank and the right image shows the 100 top ranked decoys by Zrank.

In this study, an example of a target difficult for both kinds of methods would be Siah1 (PDB ID: 4i7b). Its asymmetric unit, as well as many structural homologs, show it in a dimeric conformation, with the peptide binding at the opposite side of the protein. Additionally, there is a secondary hydrophobic groove which could maximize contacts with a potential peptide and has the same charge-distribution as the correct binding site, see Figure 6A, but which lacks the potential for *β*-sheet reinforcement seen in the true binding site. While the true site shows an average evolutionary entropy of 0.52 and the dimerization site shows a similar entropy of 0.52, the hydrophobic groove shows less conservation with an entropy of 0.54 and the rest of the protein surface shows 0.60.

For this target, InterPepRank identified several locally favorable positions for the peptide, see Figure 6B. The three wells roughly represent distances to the native peptide from decoys close to the native peptide, decoys bound at the hydrophobic groove by the helix, and decoys bound at the false dimerization site, respectively. These sites have average InterPepRank scores of 0.53, 0.50, and 0.47, respectively, while decoys bound over the rest of the protein surface average an InterPepRank score of 0.38. Similarly, the energy-based methods also report a lower average for the true binding site (−38.29 for Zrank and −3.15 for pyDock3) compared to the alternative sites (−30.45 and −3.90 for the hydrophobic groove by the helix, and 4.14 and 3.11 for the dimerization site) and the rest of the protein surface (−7.14 and 4.74 respectively), albeit with some variation as pyDock3 generally prefers the groove by the helix to the correct site and Zrank ranks the dimerization site even worse than the rest of the surface.

This demonstrates that InterPepRank does not simply select decoys arranged at conserved sites, but judges on more metrics, which is also supported by the fact that the length of the receptor multiple sequence alignment as well as the quality of alignments therein do not correlate with the quality of prediction (R < 0.1), and that simpler machine learning models with the same architecture but less edge information perform worse, as seen in the ablation study.

While in the previous example the peptide bound to Siah1 through *β*-sheet reinforcement, this represents a comparatively small fraction of the dataset (10.3% of all peptides in the dataset bind through this binding mode natively). An example of a complex with a significantly different binding mode is the c-terminal domain of Akazara scallop troponin C in complex with a fragment from the center of troponin I (PDB ID: 3tz1). The region of troponin I the peptide was isolated from is annotated as disordered by DISPROT (Piovesan et al., 2017) and the Proteus software predicts parts of it will become ordered upon binding (Basu et al., 2017). This highlights a potential difficulty in predicting the details of this interaction as the most favorable state of the peptide alone is one with a random coil, or with a transient, secondary structure. However, the experimentally solved structure of the complex shows the full peptide adopting an *α*-helical structure upon binding, which is a rather common structure for the peptides in the dataset to adopt upon binding (44.6% of the peptides in the dataset adopt a mostly *α*-helical fold when bound).

In Figure 6C, the top 100 decoys as ranked by InterPepRank and Zrank are displayed. By comparing the selection, it is evident that both methods favor the true binding site of the peptide. This example also demonstrates why Zrank has a generally lower ROC AUC than InterPepRank for most targets while at the same never scoring any target with particularly poor ROC AUCs of less than 0.3 (see Figure 3), which InterPepRank sometimes does; while Zrank prefers a particular site, it also ranks several off-site decoys favorably.

This example also highlights how InterPepRank manages to predict the correct fold of the bound peptide and incorporate that in its ranking: 96 out of the 100 best ranked decoys as ranked by InterPepRank are completely *α*-helical, while for Zrank and pyDock3 at least half the peptide decoy adopts a coil or sheet secondary structure in 41 and 27% of the top ranking decoys respectively, perhaps since a less ordered fold is energetically favorable in its unbound state or since it is easier to fit in large false positive binding sites with a high contact order as observed in the previous example.

Scatter plots for all test targets can be found online (Johansson-Åkhe et al., 2020b).

## 4 Conclusion

We have presented InterPepRank, a peptide-protein complex scoring and ranking method for use in re-scoring and selecting coarse rigid-body-docking decoys for further refinement. InterPepRank uses both the structure of the complex and evolutionary features such as PSSM and sequence conservation to achieve high accuracy scoring in manageable computational time. The structure and the features are encoded in a graph representation where physical interactions between peptide and protein are represented as edges and the features are encoded in the nodes. The graph representation is trained using graph convolutions on a large set of peptide-protein complexes to predict the quality as measured by LRMSD. To maximize performance, the outputs of an ensemble of different network architectures are averaged in the final prediction.

On a massive independent test set not used to train and validate the method, InterPepRank has a median AUC of 0.86 for finding peptide-protein complexes with LRMSD < 4Å. This is an improvement compared to other methods in the benchmark that have a median AUC of 0.65–0.79. In addition, the performance of InterPepRank is not affected by whether the peptide is disordered when unbound, which the other benchmarked methods are.

When used in the PIPER-FlexPepDock pipeline, InterPepRank consistently improves the selection of decoys for refinement, resulting in a 40% increase in high quality models produced, and a 80% increase in medium quality models produced. Additionally, by filtering poor decoys by an InterPepScore threshold, performance can reach this level without increasing the computational cost of the pipeline.

In addition to selecting peptide-protein complexes for all-atom refinement, InterPepRank should prove useful for providing a cross-target comparable scoring function.

The InterPepRank program as well as all scripts for reproducing and retraining it are available from: *http://wallnerlab.org/InterPepRank* .

## Data Availability

The original contributions presented in the study are included in the article/Supplementary Material. The finished method and all scripts necessary for reproducing and retraining it are available from: http://wallnerlab.org/InterPepRank. The dataset and additional supplementary data can be found online (Johansson-Åkhe et al., 2020b). Further inquiries can be directed to the corresponding author.
