# Supplementary material for: InterPepRank: Assessment of Docked Peptide Conformations by a Deep Graph Network
Source: Front Bioinform. 2021 Oct 25;1:763102. doi: 10.3389/fbinf.2021.763102 (PMC9581042; doi:10.3389/fbinf.2021.763102)
Supplement: Supplementary file 1 [file DataSheet1.PDF]

InterPepRank Supplementary Information

Isak Johansson Åkhe, Claudio Mirabello, Björn Wallner

1 Dataset Creation

In Figure S1, a graphical explanation of the test-, validation-, and train set creation can be found.

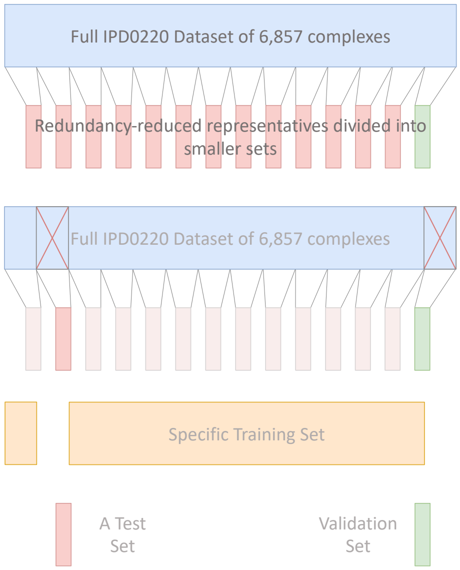

Fig. S1: A graphic for explaining the test, validation, and training set constructions. The top figure shows how the full IPD0220 dataset is redundancy-reduced by sequence similarity and further divided into the test-sets and the validation-set. The middle and bottom figure shows how a unique training set is created for each test set. The creation of the training set is simplified, as not only the complexes with sequence similarity to complexes in the test and validation sets are discarded from the potential training set, but also those which share a CATH superfamily annotation.

2 Architecture

In Tables S1-S7, S9, and S10 the detailed architectures for the 9 network architectures considered in the final ensemble for InterPepRank can be found. The nets are slight variations on the same basic architecture, as described in the main text.

**Name**	**Layer**	**Dimensions**	**Input**
Node Cons. Features	input	100×42	-
Node Ligand Var.	input	100×1	-
Amino Acid One-hot	input	100×21	-
Edge Features Input	input	100×100×4	-
Amino Acid Embed	embedding	100×4	Amino Acid One-hot
Node Features	concatenate	100×47	Amino Acid Embed<br>Node Cons. Features<br>Node Ligand Var.
Edge Features	2D dropout(25%)	100×100×4	Edge Features Input
EdgeConv1	edge conditioned convolution<br>(ReLU activation)	100×8	Edge Features<br>Node Features
EdgeConv2	edge conditioned convolution<br>(ReLU activation)	100×8	Edge Features Input<br>EdgeConv1
EdgeConv3	edge conditioned convolution<br>(ReLU activation)	100×16	Edge Features Input<br>EdgeConv2
EdgeConv4	edge conditioned convolution<br>(ReLU activation)	100×16	Edge Features Input<br>EdgeConv3
Concatenate	concatenate	100×48	EdgeConv1<br>EdgeConv2<br>EdgeConv3<br>EdgeConv4
Pooling	GlobalAveragePooling	48	Concatenate
Dropout	dropout (25%)	48	Pooling
Dense	dense	32	Dropout
Activation	ReLU activation	32	Dense
Classifier	dense	4	Activation
Output	softmax	4	Classifier

Tab. S1: Architecture for net 0 considered in the ensemble-prediction of InterPepRank. The 4 classes are evenly distributed over the range 0 to 1 as the net predicts the S-score normalized LRMSD (normalized with 4.0 LRMSD as 0.5).

Name	Layer	Dimensions	Input
Node Cons. Features	input	100×42	-
Node Ligand Var.	input	100×1	-
Amino Acid One-hot	input	100×21	-
Edge Features Input	input	100×100×4	-
Amino Acid Embed	embedding	100×4	Amino Acid One-hot
Node Features	concatenate	100×47	Amino Acid Embed<br>Node Cons. Features<br>Node Ligand Var.
Edge Features	2D dropout(25%)	100×100×4	Edge Features Input
EdgeConv1	edge conditioned convolution<br>(ReLU activation)	100×8	Edge Features<br>Node Features
EdgeConv2	edge conditioned convolution<br>(ReLU activation)	100×8	Edge Features Input<br>EdgeConv1
EdgeConv3	edge conditioned convolution<br>(ReLU activation)	100×16	Edge Features Input<br>EdgeConv2
EdgeConv4	edge conditioned convolution<br>(ReLU activation)	100×16	Edge Features Input<br>EdgeConv3
Concatenate	concatenate	100×48	EdgeConv1<br>EdgeConv2<br>EdgeConv3<br>EdgeConv4
Pooling	GlobalAveragePooling	48	Concatenate
Dropout	dropout (25%)	48	Pooling
Dense	dense	16	Dropout
Activation	ReLU activation	16	Dense
Classifier	dense	3	Activation
Output	softmax	3	Classifier

Tab. S2: Architecture for net 1 considered in the ensemble-prediction of InterPepRank. The 3 classes were the interval from 0.0 to 1.0 segmented by 0.75 and 0.5 as the net predicts the S-score normalized LRMSD (normalized with 4.0 LRMSD as 0.5).

Name	Layer	Dimensions	Input
Node Cons. Features	input	100×42	-
Node Ligand Var.	input	100×1	-
Amino Acid One-hot	input	100×21	-
Edge Features Input	input	100×100×4	-
Amino Acid Embed	embedding	100×4	Amino Acid One-hot
Node Features	concatenate	100×47	Amino Acid Embed<br>Node Cons. Features<br>Node Ligand Var.
Edge Features	2D dropout(25%)	100×100×4	Edge Features Input
EdgeConv1	edge conditioned convolution<br>(ReLU activation)	100×8	Edge Features<br>Node Features
EdgeConv2	edge conditioned convolution<br>(ReLU activation)	100×8	Edge Features Input<br>EdgeConv1
EdgeConv3	edge conditioned convolution<br>(ReLU activation)	100×16	Edge Features Input<br>EdgeConv2
EdgeConv4	edge conditioned convolution<br>(ReLU activation)	100×16	Edge Features Input<br>EdgeConv3
Concatenate	concatenate	100×48	EdgeConv1<br>EdgeConv2<br>EdgeConv3<br>EdgeConv4
Pooling	GlobalAveragePooling	48	Concatenate
Dropout	dropout (25%)	48	Pooling
Dense	dense	16	Dropout
Activation	ReLU activation	16	Dense
Classifier	dense	2	Activation
Output	softmax	2	Classifier

Tab. S3: Architecture for net 2 considered in the ensemble-prediction of InterPepRank.

Name	Layer	Dimensions	Input
Node Cons. Features	input	100×42	-
Node Ligand Var.	input	100×1	-
Amino Acid One-hot	input	100×21	-
Edge Features Input	input	100×100×4	-
Amino Acid Embed	embedding	100×2	Amino Acid One-hot
Node Features	concatenate	100×45	Amino Acid Embed<br>Node Cons. Features<br>Node Ligand Var.
Edge Features	2D dropout(25%)	100×100×4	Edge Features Input
EdgeConv1	edge conditioned convolution<br>(ReLU activation)	100×8	Edge Features<br>Node Features
EdgeConv2	edge conditioned convolution<br>(ReLU activation)	100×8	Edge Features Input<br>EdgeConv1
EdgeConv3	edge conditioned convolution<br>(ReLU activation)	100×16	Edge Features Input<br>EdgeConv2
EdgeConv4	edge conditioned convolution<br>(ReLU activation)	100×16	Edge Features Input<br>EdgeConv3
Concatenate	concatenate	100×48	EdgeConv1<br>EdgeConv2<br>EdgeConv3<br>EdgeConv4
Pooling	GlobalAveragePooling	48	Concatenate
Dropout	dropout (25%)	48	Pooling
Dense	dense	32	Dropout
Activation	ReLU activation	32	Dense
Classifier	dense	2	Activation
Output	softmax	2	Classifier

Tab. S4: Architecture for net 3 considered in the ensemble-prediction of InterPepRank.

Name	Layer	Dimensions	Input
Node Cons. Features	input	100×42	-
Node Ligand Var.	input	100×1	-
Amino Acid One-hot	input	100×21	-
Edge Features Input	input	100×100×4	-
Amino Acid Embed	embedding	100×4	Amino Acid One-hot
Node Features	concatenate	100×47	Amino Acid Embed<br>Node Cons. Features<br>Node Ligand Var.
Edge Features	2D dropout(10%)	100×100×4	Edge Features Input
EdgeConv1	edge conditioned convolution<br>(kernel net 8, ReLU activation)	100×8	Edge Features<br>Node Features
EdgeConv2	edge conditioned convolution<br>(kernel net 8, ReLU activation)	100×8	Edge Features Input<br>EdgeConv1
EdgeConv3	edge conditioned convolution<br>(kernel net 16, ReLU activation)	100×16	Edge Features Input<br>EdgeConv2
EdgeConv4	edge conditioned convolution<br>(kernel net 16, ReLU activation)	100×16	Edge Features Input<br>EdgeConv3
Concatenate	concatenate	100×48	EdgeConv1<br>EdgeConv2<br>EdgeConv3<br>EdgeConv4
Pooling	GlobalAveragePooling	48	Concatenate
Dropout	dropout (10%)	48	Pooling
Dense	dense	32	Dropout
Activation	ReLU activation	32	Dense
Classifier	dense	2	Activation
Output	softmax	2	Classifier

Tab. S5: Architecture for net 4 considered in the ensemble-prediction of InterPepRank.

Name	Layer	Dimensions	Input
Node Cons. Features	input	50×42	-
Node Ligand Var.	input	50×1	-
Amino Acid One-hot	input	50×21	-
Edge Features Input	input	50×50×4	-
Amino Acid Embed	embedding	50×4	Amino Acid One-hot
Node Features	concatenate	50×47	Amino Acid Embed<br>Node Cons. Features<br>Node Ligand Var.
Edge Features	2D dropout(10%)	50×50×4	Edge Features Input
EdgeConv1	edge conditioned convolution<br>(ReLU activation)	50×8	Edge Features<br>Node Features
EdgeConv2	edge conditioned convolution<br>(ReLU activation)	50×8	Edge Features Input<br>EdgeConv1
EdgeConv3	edge conditioned convolution<br>(ReLU activation)	50×16	Edge Features Input<br>EdgeConv2
EdgeConv4	edge conditioned convolution<br>(ReLU activation)	50×16	Edge Features Input<br>EdgeConv3
Concatenate	concatenate	50×48	EdgeConv1<br>EdgeConv2<br>EdgeConv3<br>EdgeConv4
Pooling	GlobalAveragePooling	48	Concatenate
Dropout	dropout (10%)	48	Pooling
Dense	dense	32	Dropout
Activation	ReLU activation	32	Dense
Classifier	dense	2	Activation
Output	softmax	2	Classifier

Tab. S6: Architecture for net 5 considered in the ensemble-prediction of InterPepRank. The input for this net was constructed the same way as for the other networks, but with a limit of 50 residues rather than 100.

**Name**	**Layer**	**Dimensions**	**Input**
Node Cons. Features	input	100×42	-
Node Ligand Var.	input	100×1	-
Amino Acid One-hot	input	100×21	-
Node Features	concatenate	100×64	Node Cons. Features<br>Node Ligand Var.<br>Amino Acid One-hot
Edge Features	input	100×100×4	-
EdgeConv1	edge conditioned convolution<br>(kernel net 8, ReLU activation)	100×8	Edge Features<br>Node Features
BatchNorm1	batch normalization	100×8	EdgeConv1
Dropout1	dropout (10%)	100×8	BatchNorm1
EdgeConv2	edge conditioned convolution<br>(kernel net 8, ReLU activation)	100×8	Edge Features<br>Dropout1
BatchNorm2	batch normalization	100×8	EdgeConv2
Dropout2	dropout (10%)	100×8	BatchNorm2
Bypass1	1D dense	100×8	Node Features
Block1	addition	100×8	Dropout2<br>Bypass1
EdgeConv3	edge conditioned convolution<br>(kernel net 8, ReLU activation)	100×8	Edge Features<br>Block1
BatchNorm3	batch normalization	100×8	EdgeConv3
Dropout3	dropout (10%)	100×8	BatchNorm3
EdgeConv4	edge conditioned convolution<br>(kernel net 8, ReLU activation)	100×8	Edge Features<br>Dropout3
BatchNorm4	batch normalization	100×8	EdgeConv4
Dropout4	dropout (10%)	100×8	BatchNorm4
Block2	addition	100×8	Dropout4<br>Block1
EdgeConv5	edge conditioned convolution<br>(kernel net 8, ReLU activation)	100×8	Edge Features<br>Block2
BatchNorm5	batch normalization	100×8	EdgeConv5
Dropout5	dropout (10%)	100×8	BatchNorm5
EdgeConv6	edge conditioned convolution<br>(kernel net 8, ReLU activation)	100×8	Edge Features<br>Dropout5
BatchNorm6	batch normalization	100×8	EdgeConv6
Dropout6	dropout (10%)	100×8	BatchNorm6
Block3	addition	100×8	Dropout6<br>Block2
EdgeConv7	edge conditioned convolution<br>(kernel net 8, ReLU activation)	100×8	Edge Features<br>Block3
BatchNorm7	batch normalization	100×8	EdgeConv7
Dropout7	dropout (10%)	100×8	BatchNorm7
EdgeConv8	edge conditioned convolution<br>(kernel net 8, ReLU activation)	100×8	Edge Features<br>Dropout7
BatchNorm8	batch normalization	100×8	EdgeConv8
Dropout8	dropout (10%)	100×8	BatchNorm8
Block4	addition	100×8	Dropout8<br>Block3
EdgeConv9	edge conditioned convolution<br>(kernel net 8, ReLU activation)	100×32	Edge Features<br>Block4
Pooling	GlobalAttentionPool	32	EdgeConv9
BatchNorm9	batch normalization	32	EdgeConv9
Dropout9	dropout (10%)	32	BatchNorm9
Prediction	dense	1	Dropout9

Tab. S7: Architecture for net 6 considered in the ensemble-prediction of InterPepRank. Training of net 6 was done in a binary connected manner, running two copies of the net in parallel with weight-sharing inbetween on two different decoys at any given moment. Additionally, during training another net found in Table S8 was attached to the binary net, and the loss function was calculated on this net's capacity to classify which of the two decoys is closer to native, as well as the individual losses from the single branches, weighting single branches 0.1 and the comparison at 1.0. This approach is similar to the Tricephalous net suggested by Hurtado *et al.* (2018).

Name	Layer	Dimensions	Input
EdgeConv10	edge conditioned convolution<br>(kernel net 16, ReLU activation)<br>weight-sharing with EdgeConv11	100×16	Edge Features 6_1<br>Block4 6_1
EdgeConv11	edge conditioned convolution<br>(kernel net 16, ReLU activation)<br>weight-sharing with EdgeConv10	100×16	Edge Features 6_2<br>Block4 6_2
EdgeConv12	edge conditioned convolution<br>(kernel net 16, ReLU activation)<br>weight-sharing with EdgeConv13	100×16	Edge Features 6_1<br>Block4 6_1
EdgeConv13	edge conditioned convolution<br>(kernel net 16, ReLU activation)<br>weight-sharing with EdgeConv12	100×16	Edge Features 6_2<br>Block4 6_2
Pooling1	GlobalAttentionPool (32)	32	EdgeConv10
Pooling2	GlobalAttentionPool (32)	32	EdgeConv11
Pooling3	GlobalAttentionPool (32)	32	EdgeConv12
Pooling4	GlobalAttentionPool (32)	32	EdgeConv13
Add1	addition	32	Pooling1<br>Pooling4
Add2	addition	32	Pooling2<br>Pooling3
BatchNorm10	batch normalization	32	Add1
Dropout10	dropout (10%)	32	BatchNorm10
BatchNorm11	batch normalization	32	Add2
Dropout11	dropout (10%)	32	BatchNorm11
Dense1	dense	1	Dropout10
Dense2	dense	1	Dropout11
Comparison	addition	1	Dense1<br>Dense2

Tab. S8: The extra comparison-net used during training of net 6, attached to two weight-sharing instances of net 6 referred to as net 6_1 and 6_2.

Name	Layer	Dimensions	Input
Node Cons. Features	input	100×42	-
Node Ligand Var.	input	100×1	-
Amino Acid One-hot	input	100×21	-
Edge Features Input	input	100×100×4	-
Amino Acid Embed	embedding	100×4	Amino Acid One-hot
Node Features	concatenate	100×47	Amino Acid Embed<br>Node Cons. Features<br>Node Ligand Var.
Edge Features	2D dropout(25%)	100×100×4	Edge Features Input
EdgeConv1	edge conditioned convolution<br>(kernel net 8, ReLU activation)	100×8	Edge Features<br>Node Features
EdgeConv2	edge conditioned convolution<br>(kernel net 8, ReLU activation)	100×8	Edge Features<br>EdgeConv1
EdgeConv3	edge conditioned convolution<br>(kernel net 16, ReLU activation)	100×16	Edge Features<br>EdgeConv2
EdgeConv4	edge conditioned convolution<br>(kernel net 16, ReLU activation)	100×16	Edge Features<br>EdgeConv3
Concatenate	concatenate	100×48	EdgeConv1<br>EdgeConv2<br>EdgeConv3<br>EdgeConv4
Pooling	GlobalAttentionPooling	32	Concatenate
Dropout	dropout (25%)	32	Pooling
Dense	dense	32	Dropout
Activation	ReLU activation	32	Dense
Classifier	dense	4	Activation
Output	softmax	4	Classifier

Tab. S9: Architecture for net 7 considered in the ensemble-prediction of InterPepRank. The 4 classes are evenly distributed over the range 0 to 1 as the net predicts the S-score normalized LRMSD (normalized with 4.0 LRMSD as 0.5).

Name	Layer	Dimensions	Input
Node Cons. Features	input	100×42	-
Node Ligand Var.	input	100×1	-
Amino Acid One-hot	input	100×21	-
Edge Features Input	input	100×100×4	-
Amino Acid Embed	embedding	100×4	Amino Acid One-hot
Node Features	concatenate	100×47	Amino Acid Embed<br>Node Cons. Features<br>Node Ligand Var.
Edge Features	2D dropout(10%)	100×100×4	Edge Features Input
EdgeConv1	edge conditioned convolution<br>(kernel net 8, ReLU activation)	100×8	Edge Features<br>Node Features
EdgeConv2	edge conditioned convolution<br>(kernel net 8, ReLU activation)	100×8	Edge Features<br>EdgeConv1
EdgeConv3	edge conditioned convolution<br>(kernel net 16, ReLU activation)	100×16	Edge Features<br>EdgeConv2
EdgeConv4	edge conditioned convolution<br>(kernel net 16, ReLU activation)	100×16	Edge Features<br>EdgeConv3
Concatenate	concatenate	100×48	EdgeConv1<br>EdgeConv2<br>EdgeConv3<br>EdgeConv4
Pooling	GlobalAttentionPooling	32	Concatenate
Dropout	dropout (10%)	32	Pooling
Dense	dense	32	Dropout
Activation	ReLU activation	32	Dense
Classifier	dense	2	Activation
Output	softmax	2	Classifier

Tab. S10: Architecture for net 8 considered in the ensemble-prediction of InterPepRank.

2.1 Discussion on Architecture Performance

Most of the final network architectures performed similarly to each other, see Figure S2, which is unsurprising considering most of them are based on the same general architecture framework. The outlier of network 5 (Table S6) can be explained by its small input size, only 50 considered residues compared to 100 for the other architectures. For some particularly large peptides in the test sets, this means that the entire interaction-surface might not even be covered. However, increasing the number of nodes up to 250 and above did not significantly change the performance (data not shown). A more surprising difference in performance is between network number 8 (Table S10) and 7 (Table S9). Network architecture 8 is virtually identical to architecture 7, with the differences of a less aggressive dropout during training and being a 2-class classifier rather than a 4-class classifier. In fact, most of the networks acting as a multi-classifier rather than a 2-class classifier shows markedly better performance, which can probably be attributed to the fact that they have access to more data regarding the targets during training, considering the more fine-grained representation of the loss.

Networks 7 and 8 also implement a global attention pooling for their pooling layers, rather than a global average pooling, but this change did not seem to translate to any significant improvement in performance. Attention layers perform best with large amounts of varied data with inherent patterns, such as text decoding or recognition of protein motifs in sequences, and it is possible that their performance is limited here by the relatively low number of truly unique positive decoy structures. All peptide-protein complex decoys are derived from no more than 6,587 different complexes, and all positive decoys in the data set are by definition structures which are similar to these, limiting the variation of the data that can be learned.

This relatively low variance between positive decoys might also be a contributing factor to why network architecture 6 did not achieve better performance, even with a much deeper architecture and a learning scheme previously shown to facilitate comparison in quality assessment. However, another reason for this might be the small size of the interim layers, kept down by necessity to keep the speed of the network reasonable for the evaluation of thousands of decoys in minutes. As shown in Li *et al.* (2019), using residual couplings as done in this work can allow for a considerably deeper network, but this might not always lead to a boost in performance, especially not when not coupled with wide layers and dilated convolutions.

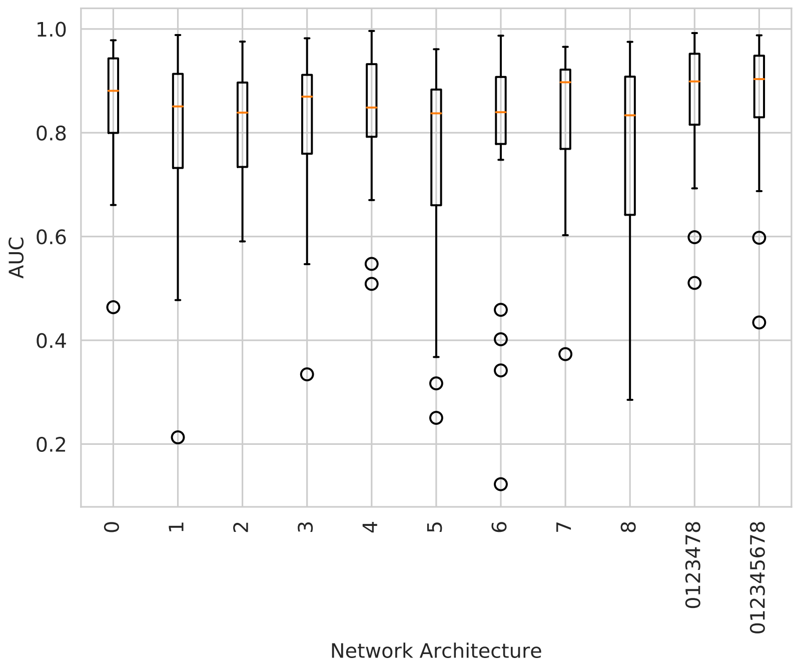

Fig. S2: AUC on validation targets for the different individual network architectures, the final ensemble method and an ensemble including all architectures. The network architectures are numbered from 0 through 8, and the ensemble methods are named after the included architectures. The ensemble 0123478 shows optimal performance on the validation data. A detailed description of the architectures and their differences can be found in the Supplementary Information.

3 Precision-Recall Curves

For every ROC-curve in the main paper, Figure S3 includes a corresponding Precision-Recall curve.

ROC-curves measure predictive power independently of the bias in data set labeling, making them suitable for cross-target comparison where different peptide-protein target pairs yield different distributions of LRMSD of their decoy sets because of parameters such as receptor size, peptide size, or general success or bias of the docking method, and making analysis of heavily biased data sets easier. Precision-Recall curves on the other hand provide an absolute metric of performance. Because of the inherently different distributions of LRMSD of decoys generated from different target pairs, the individual curves in the sub figures of Figure S3 are not comparable to each other, but they are included here as a frame of reference of what kind of performance can be expected when applying the analyzed methods on decoys generated by fast-fourier docking.

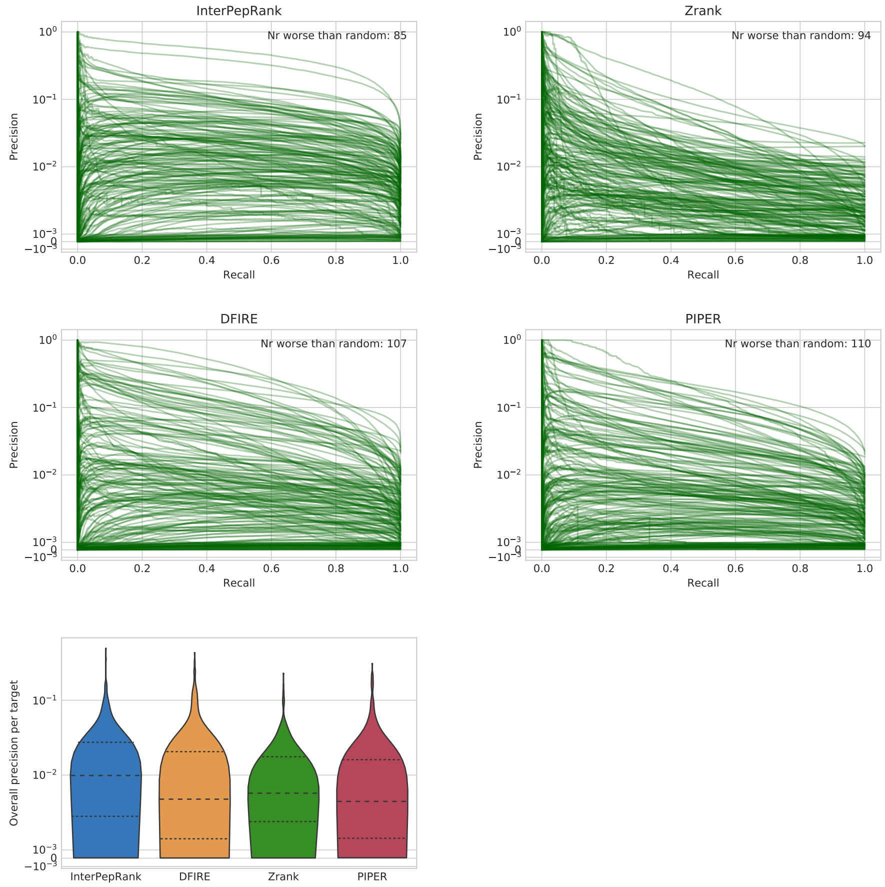

Fig. S3: Precision-recall-curves for the different methods, each target is represented by 1 curve, and a violin-plot over the distributions of AUCs (average percisions). The area under the curve (AUC) displayed in the graphs is the average and median over all targets.

4 Disordered Peptides

The full set of complexes in the dataset was divided into a ”disordered” and ”ordered” set based on how much of the peptide in each complex was predicted to be disordered when unbound by DISOPRED Ward *et al.* (2004). A peptide was considered disordered if at least 75% of it was predicted as disordered when unbound. Conversely, a peptide was considered ordered if no more than 10% of it was predicted as disordered when unbound. Differences in AUC distribution is visualized in Figure S4.

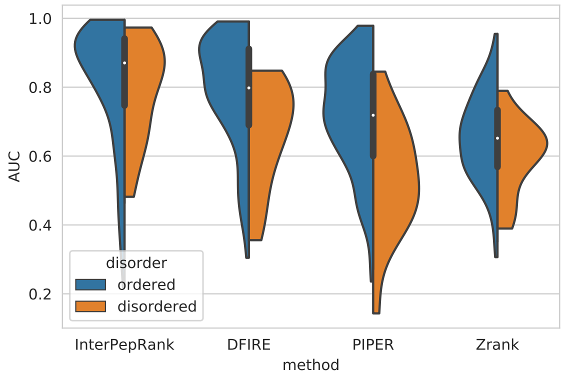

Fig. S4: Differences in AUC distribution for peptides predicted to be disordered when unbound and those predicted to be ordered when unbound.

5 Expanded Analysis test set

With a decrease in test set size, computationally heavy re-scoring methods like pyDock3 or Rosetta FlexPepDock scoring-mode can be included in the comparison. See Figure S5 for an analogue to Figure 4 of the main paper.

Using Rosetta FlexPepDock scoring mode only to re-score rigid-body docked decoys proved slow, even without any refinement, as was discussed in the main paper. Since the Rosetta scoring function is a fine-grained function developed for protein refinement and design, it makes sense it would perform poorly on structures not necessarily optimal by the Rosetta standard. Indeed, when using Rosetta to score structures, it is common practice to first relax the structure through the Rosetta Relax protocol, something which would considerably add to the run-time if attempted in this situation.

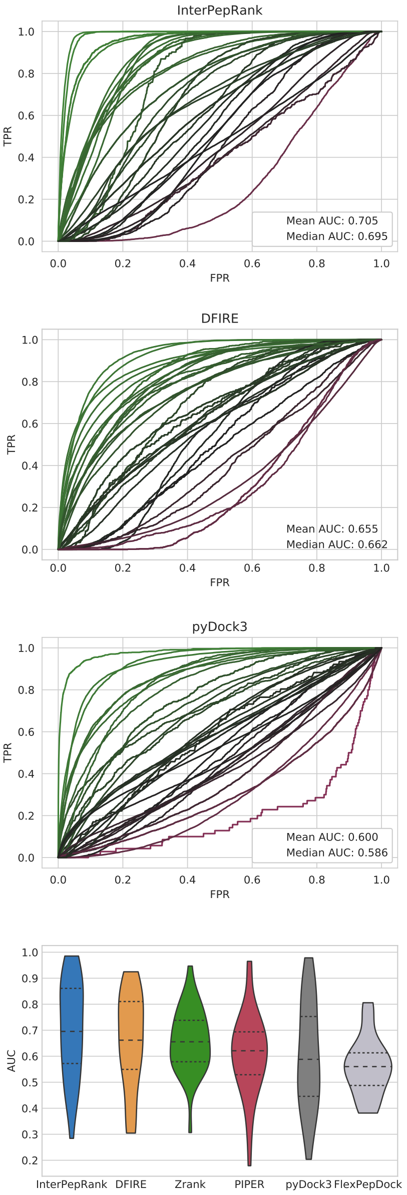

Fig. S5: ROC-curves for all methods discussed in the main paper, including pyDock3, and a violin-curve summarizing all AUCs, for the Expanded Analysis set (a randomly selected set of 50 targets all methods were run on). Each target is represented by 1 curve. The area under the curve (AUC) displayed in the graphs is the average and median over all targets.

6 Decoy Distribution

In Figure S6 are some graphical representations of the LRMSD distributions of decoys selected by the different scoring methods for refinement. Results are only shown for the set all methods were run on.

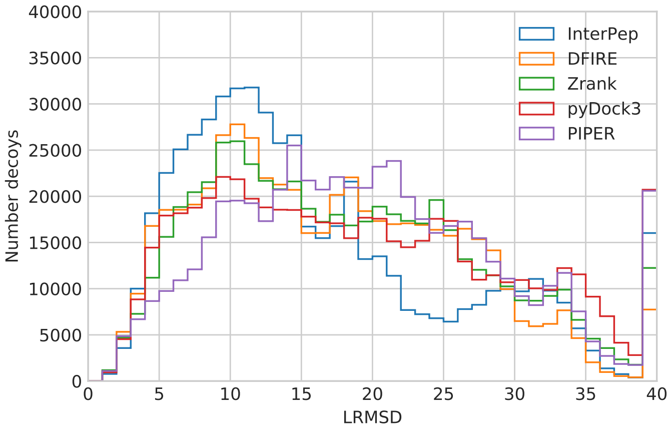

(a)

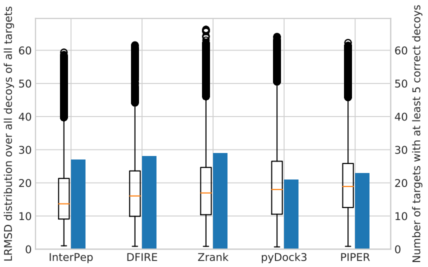

(b)

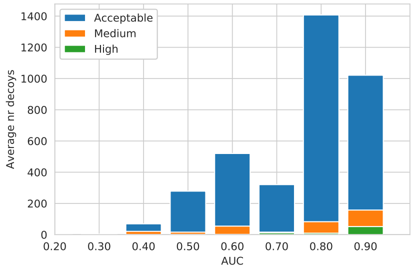

(c)

Fig. S6: Distribution of LRMSD of decoys selected for refinement by the different methods. Results shown for the Expanded Analysis set. In (a), all decoys at LRMSD 40+ were summed into the 40 Å bin. In (c), the median number of models of the different quality-measures produced per method per target after refinement for all methods and targets in the Expanded Analysis set are shown binned by AUC on original decoys, to highlight that good performance on the rigid-body docked decoys translates to well-refined models.

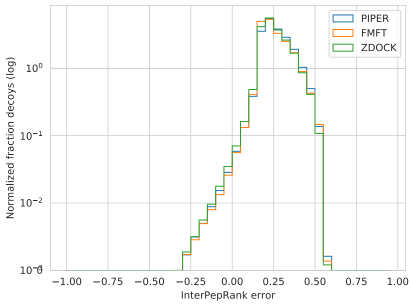

(a)

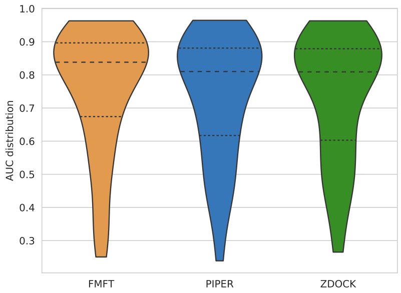

(b)

Fig. S7: (a) Distribution of InterPepRank errors for decoys generated by different docking algorithms for the Expanded Analysis Set. (b) Distribution of AUCs for InterPepRank scoring of decoys generated by different docking algorithms for the Expanded Analysis Set.

7 Bias Towards Different Docking Algorithms

Figure S7 shows the distribution of error of InterPepRank over decoys generated by different docking algorithms.

8 Interface complexity bias

As can be seen in Figure S8, all investigated methods except for InterPepRank shows a slight decrease in performance when the contact order of the true binding site is low. This indicates that if the binding site consists only of continuous stretches of the receptor, as is the case with β-sheet reinforcement, the other methods investigated will see a small but significant decrease in performance. In fact, if only interfaces with high-contact-order native peptide-binding interfaces are investigated, the performances of DFIRE and InterPepRank cannot be said to be significantly different.

Contact order is here calculated by, for each residue in a given set of residues, calculating the average distances between these residues in sequence. Any distance above 10 is counted as 10 instead. For example, if we calculate the contact order for a set of residues positioned along one side of an α-helix, that set would then have a contact order of approximately 3.6 as there are around 3.6 residues per turn in most α-helices.

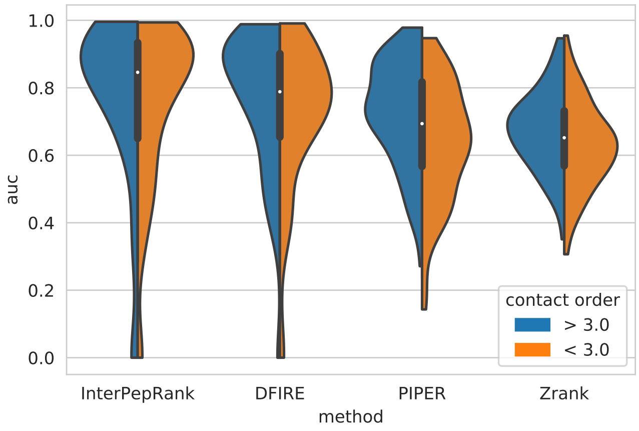

Fig. S8: Distribution of AUCs for different targets separated by whether the true binding site has a contact order larger than or equal to 3 or not. This cutoff was selected as an interface encompassing one side of a helix (similar to a coiled coil structure) would yield a contact order of slightly above 3 and interfaces mainly composed of β-sheet reinforcement would yield a contact order of around 1.

9 Remote Homologs and Similar Interface

The test, validation, and training sets are separated by both sequence identity as well as CATH superfamily of the receptor. Still, there exists a possibility for remote sequence homologs being present in the training set, undetectable by sequence identity. Additionally, while two proteins belonging to the same CATH superfamily means they share overall topological features in excess of a demonstrable evolutionary relationship, this does not necessarily mean that two proteins which do not share CATH superfamily cannot share some structural motifs, such as similar interfaces.

The core idea of a machine learning method is that it should be able to make use of and generalize over remote differences, but a problem arises if, rather than finding a general solution, the machine learning algorithm simply stores training target information like a look-up table. In this case, rather than demonstrating generalizability over novel datapoints, a machine learning algorithm would in effect work like a template based method or simple k-nearest-neighbors approach where it can look through its stored look-up table for the most similar previous case and use it as a reference. If a machine learning method has failed to generalize, its performance will risk being over-estimated when the test set contains targets with remote similarity to individual targets in the train set.

Similarities between targets in the test and train sets were investigated to

infer whether InterPepRank was truly able to generalize over targets or if the information stream between sets was too great, which would result in InterPepRank working like a template-based method and not being able to generalize to novel complexes. To this end, every receptor in every test set was compared to all receptors of their respective training sets.

9.1 PSSM-PSSM similarity

Firstly, the PSSM-profiles of all test targets were pairwise aligned to all training data PSSM using palignp Ohlson *et al.* (2004) and E-values were generated by fitting Gumbel-distributions to the score distributions of aligning random PSSM for every target, similarly to Gao and Skolnick (2010), and adjusting for database size. By this analysis, no test target had any PSSM-PSSM significant match (E-value $< 10^{-3}$) in its training set.

9.2 Interface similarity

Secondly, the interfaces of every test complex was compared to every train complex using iAlign (Gao and Skolnick (2010)). In this case, 10 targets in the test set matched to different targets in their training sets with E-values less than or equal to 10^{-3}, indicating that for these 10 out of 687 targets, there was a template for interaction available in its training set for use in a k-nearest-neighbor like approach. However, the ROC AUC for InterPepRank to select correct decoys for each of these 10 targets was not significantly different from its performance on targets without any such similarities (p-value > 0.17), nor could any correlation between interface similarity between a target and its training data and InterPepRank performance be proven (p-value > 0.67).

9.3 Representative Set Annex

In Table S11, the 687 peptide-protein complex representatives utilized as true native structures in this study can be found.

1aw8BA	1bc5AT	1bgyJK	1cmxCD	1cqtBJ	1czyAD	1d4wAC	1dkdDH	1e91AB
1eakDR	1eg4AP	1ejhAE	1elwAC	1emuAB	1f47BA	1f59AC	1g0yRI	1gagAB
1gxcDE	1gy3CF	1hesAP	1hqqCG	1hr8HR	1htmFE	1i4oAC	1i51DF	1ilqAC
1isqAB	1j2xAB	1jd6AB	1jdpAH	1jm4BA	1jmtAB	1jw6AB	1kcrLP	1ky7AP
1lj2AC	1ljzAB	1m06FJ	1mk9DA	1n7fBD	1nltAB	1npqAB	1nx0AC	1o0pAB
1om9BQ	1ozsAB	1p16BD	1pu9AB	1q0wBA	1qd6DB	1rfiAC	1rpqAW	1rxmAB
1szaBZ	1t0jBC	1t2vAF	1tn6BC	1tqeQX	1tt5BE	1ttwAB	1twbAC	1ty4BD
1vf5CG	1vppVX	1w70AC	1yrkAB	1ywhMN	1yypAB	1z3mES	1z7z34	1z9oDJ
1zkkBF	2a40AC	2a4jAB	2a7uBA	2aucCD	2buoAT	2c5iTP	2cciDI	2ce9AX
2cnmAD	2cnzAB	2dohXC	2dvqBQ	2dymAB	2f69AB	2f9uAB	2fffBA	2fmkAB
2fymDE	2g46AC	2ggmBD	2ghtBD	2h1cAB	2hdxEK	2hu2AB	2hwf14	2hzsFK
2ibfAD	2ifrAB	2isqAB	2j2897	2jbyAB	2jdlAC	2jktIQ	2jmjAP	2jz3CA
2k17AP	2k9uAB	2kbmAX	2knhAB	2kqfAB	2kqsAB	2krbAB	2ks9AB	2kxcAB
2kxqAB	2kzuAB	2l0yAB	2l1cAB	2l6eAB	2lbmAC	2lcsAB	2lctAB	2lozAB
2lp0AB	2lskAB	2lspBA	2lsrAB	2lsvAB	2lxsAB	2makAB	2mc6AB	2mkcAB
2mnjBA	2mowAB	2mv7AB	2mwoAB	2mzdAB	2n01BA	2n0yAB	2n3kAB	2nnuAB
2ns8AE	2nudBD	2p0wBQ	2p5bAI	2pehAC	2phkAB	2pieAF	2pnxCD	2pqnAB
2pv2BE	2pv3AC	2pvcBD	2q6gAC	2qasAB	2qiyAC	2qmeAI	2qqgAB	2qqsAD
2r9qBY	2rquAB	2rqwAB	2rr4AB	2rt5AB	2v2fFA	2v86BD	2v8fBC	2vdoAC
2vzgBA	2w2uBD	2w6jHI	2w84AB	2wa8AB	2wo6BC	2x04BD	2x39AC	2x4yOP
2xc8BC	2xjzCK	2xpoCD	2xpxAB	2xqnTA	2xs0AB	2xvcAB	2xxnAB	2xzeBR
2y65CW	2ybfAB	2ykaAB	2yktAB	2z3fCK	2zjdAB	3a0ben	3aaeIV	3aaeJV
3agzAC	3al3AB	3aloAE	3aslAB	3auwBA	3bimDK	3bqoAB	3brfAD	3bzxBA
3c01HD	3c0tAB	3chxJL	3cxwAB	3cyyAD	3d1fBQ	3d8aDS	3d9nBZ	3dabEF
3dd7AB	3dktCM	3e1kEF	3e2bAC	3e2uCG	3echBC	3ehuAC	3er5EI	3eu7AX
3eyfBE	3f1iSC	3f2oAC	3fdlAB	3fksPR	3fxxAB	3g2uAC	3g7lAP	3g7zAC
3ggzBF	3gjoCG	3gl6AB	3gm1AE	3gz1AQ	3h1zAP	3h52AN	3hdiBD	3hymHG
3i5rAB	3iaxAB	3iciAC	3ik5AB	3iswAC	3izoBG	3j47VQ	3j47US	3jc21w
3jq5AB	3jqolm	3juaGH	3jwrBD	3k48AS	3kzeCE	3l6yAB	3lcnAC	3lgeBF
3liyEK	3lm1IJ	3mazAB	3mk4AB	3ml4CG	3mpjBY	3n5eBD	3na1BD	3nk4AC
3nmxBE	3o0eFQ	3o42AB	3oe0AI	3olrAE	3oszAB	3p72AB	3pbpDF	3pcsAE
3pe4AB	3pluAC	3pqrAB	3q47BC	3q6sCE	3qbrAB	3qisAB	3qksAC	3r0hDd
3r42AB	3r7gAB	3r9iCH	3rqeCE	3rqfAE	3sj9AB	3sl9BD	3swcAP	3t7gAC
3tdiBC	3tduAF	3tdzBF	3twwBD	3tz1AB	3tzxBC	3u1jBA	3u5nAC	3u7dCD
3ubwAP	3ueoDE	3um2AB	3upvAB	3vivBC	3w6kCA	3wbnAB	3wp1BA	3wuuAC
3wxaAC	3zfwBY	3zhaDJ	3zilAB	3zrjAX	3zrzAC	4a1gDH	4a2aBD	4a62AC
4ajyVH	4aktAC	4am9AB	4au7AC	4b45AB	4b60AC	4bh6GO	4bj6CD	4bl0DF
4bq6FE	4bqdBD	4btaAC	4bu1AC	4bwsDE	4bxrAC	4bxwAF	4c1qAC	4c31EF
4c5iAC	4cc9BC	4ccoBD	4cfhBC	4chbBD	4cugBF	4cydBF	4dayAC	4dcbAF
4dowAC	4dxrAB	4eqfAB	4ezvAC	4f02DF	4fbwBD	4fifAC	4flnBE	4fsjBE
4g2vAB	4geqCE	4gneAB	4gq6AB	4gxlAB	4h0hBD	4h2tAC	4h3hEF	4h62QK
4hrhAC	4htpAC	4i7bAB	4igaAB	4iimAC	4ikaAD	4imiDF	4irvBF	4isrBE
4iuuBC	4j1vCF	4j2cCD	4j2lAC	4j8sAB	4jhkAC	4jifAB	4jmhAB	4jmrCH
4jo8AB	4jolDH	4jqiAV	4k0uAB	4l1uCH	4l7xAU	4lebAB	4lk9AB	4lnpAB
4m5sAB	4m6bDF	4mi8BD	4mliAB	4mzgBA	4mzjAT	4n4hAB	4nawAD	4nb3AC
4nf9BD	4nuvAC	4obhCF	4od7AD	4odlAD	4oi4CD	4oucAB	4oykAC	4oz1BC
4pi0KN	4piqAB	4psiBE	4pyuAC	4q5uAC	4q96EC	4qaeFU	4qbrCE	4qeoAP
4qmfDC	4qmgEJ	4qxbAE	4r4nAa	4reyAB	4riqDF	4rqiBF	4rrvAB	4s0rLS
4tk4BC	4tknAD	4tmpAB	4tt3EJ	4tvqCE	4txqBD	4tzoEF	4u39GP	4u6yAP
4u7iAB	4uf1AB	4um9DF	4uqyAB	4uwxAC	4ux6BA	4w4zBF	4w5aBD	4wj7AW
4wjpAB	4wphBC	4wsfAB	4wzxAE	4x2oAC	4x3hAB	4x8pAB	4xevAC	4xgrGH
4xtrAG	4xvjHA	4yl6AB	4yosAE	4ywcBD	4yzhAB	4z0rBD	4z2pBC	4zbjBD
4znyAB	4zoxAB	4zozAY	4zqwBA	5a53AB	5a5bRY	5aerAC	5ajkCD	5awuAB
5b4wDJ	5c5eAH	5c7fDH	5cqyAC	5cvdBE	5dahAD	5de2AC	5di8AC	5dxaAF
5e4wDF	5efiAC	5eftBA	5en7CD	5ev0BD	5f0pBD	5f5vDF	5f67AC	5fgcEA
5fjwAL	5fn3BG	5fpxBF	5frqAG	5ft1GH	5fvlBC	5fw5AC	5fztAB	5gk9AB
5glfCD	5gowBA	5gtbAB	5gtuAB	5h5qAB	5h7yAB	5h9dBL	5hawBK	5hkhAB
5hkyAB	5hoiAD	5hvzAC	5i22AB	5ig9AI	5ii0CF	5itzBD	5ix2BQ	5ixdAB
5j3hEB	5jcyAB	5jelAB	5jmeBF	5jnbDH	5jtpCG	5k2mIM	5kc1LK	5koaBD
5l0yCI	5lasBD	5ldeBR	5lhzBE	5lm1AB	5lmgAD	5lmxGD	5lsjCQ	5ly3AB
5m0jCI	5m5sBH	5m72AB	5m9eAE	5mf9AB	5mfgAE	5mgxFA	5mk1CH	5mu3BC
5mx2zy	5n22AE	5nc7AI	5nl1AH	5oakCD	5ojrAE	5ok6BC	5okzGJ	5onbAB
5onsAB	5oo6TU	5ovpAB	5oxwAH	5supBG	5swfAB	5t0qAB	5tgiBD	5tzqBC
5u1gDK	5u1mAB	5ua5AB	5v1dCF	5v1tAB	5v90CD	5va6AC	5vaoAF	5vb9BD
5vklAB	5vmoAB	5vwvAB	5vzuBE	5w2jBF	5w4sAB	5w93AD	5wa4AM	5waiBD
5wbhDW	5wggAB	5wknBD	5wriAD	5wtbAE	5wvoCD	5xfqBF	5xjr2M	5xodAB
5xpuAB	5xsqCD	5xtbIW	5xtbQW	5xvwEF	5xxfAE	5xyfAB	5y59BC	5y7wBD
5yc4AP	5yf4AB	5ygfAD	5yt0AB	5zfuBH	5znrBQ	5zooGA	5zt0BI	5zz9AE
6a5eDF	6ak2AD	6am0AC	6am0BC	6b0xDd	6b2zMT	6b3xAB	6b9hAB	6bheAB
6c0aAB	6cnlGS	6d6rFJ	6dcnAD	6deiAC	6drtBE	6ef31n	6eiwBD	6et51y
6f6dAB	6f8gAE	6fbkAP	6fkqAB	6fo2BI	6g0yAJ	6g84BC	6gbeAB	6gd5AB
6gfqBC	6gos1A	6gqnBC	6gvlAB	6gy2AD	6h41AB	6h9jAD	6h9oCD	6ha6AD
6hboAB	6he5GL	6hksAG	6hosBC	6hquFN	6hy2XA	6i5nAI	6ie4AB	6ituAB
6iuiAD	6ixpDE	6j0wAC	6j6hTn	6j8nBA	6jjzBD	6jx3BA	6mhfAC	6mlcCF
6n3eAB	6n87AC	6n9xAF	6nj8CF	6njgCB	6nnvCK	6ocpCP	6oi4AE	6oswAB
6punCF	6q36BD	6qcgAH	6qnpCJ	6qxfMN	6rh6AB	6rr0BI	6rrcCB	6s07AC
6s1rAB	6s3fAB	6s8nFC						

Tab. S11: 687 peptide-protein complexes.

References

Gao, M. and Skolnick, J. (2010). ialign: a method for the structural comparison of protein–protein interfaces. *Bioinformatics*, **26**(18), 2259–2265.

Hurtado, D. M., Uziela, K., and Elofsson, A. (2018). Deep transfer learning in the assessment of the quality of protein models. *arXiv preprint arXiv:1804.06281*.

Li, G., Muller, M., Thabet, A., and Ghanem, B. (2019). Deepgcns: Can gcns go as deep as cnns? In *Proceedings of the IEEE/CVF International Conference on Computer Vision*, pages 9267–9276.

Ohlson, T., Wallner, B., and Elofsson, A. (2004). Profile–profile methods provide improved fold-recognition: A study of different profile–profile alignment methods. *Proteins: Structure, Function, and Bioinformatics*, **57**(1), 188–197.

Ward, J. J., Sodhi, J. S., McGuffin, L. J., Buxton, B. F., and Jones, D. T. (2004). Prediction and functional analysis of native disorder in proteins from the three kingdoms of life. *Journal of molecular biology*, **337**(3), 635–645.
